# Disturbance frequency directs microbial community succession in marine biofilms exposed to shear

**DOI:** 10.1128/msphere.00248-23

**Published:** 2023-10-16

**Authors:** Abhishek T. Naik, Kristina M. Kamensky, Aren M. Hellum, Pia H. Moisander

**Affiliations:** 1 Department of Biology, University of Massachusetts Dartmouth, North Dartmouth, Massachusetts, USA; 2 School of Marine Science and Technology, University of Massachusetts Dartmouth, New Bedford, Massachusetts, USA; 3 Naval Undersea Warfare Center, Newport, Rhode Island, USA; Clemson University, Clemson, South Carolina, USA

**Keywords:** marine biofilm, shear, community succession, biofouling, disturbance

## Abstract

Marine biofilm growth poses significant challenges across marine industries (“biofouling”). Understanding the microbial communities involved in biofouling processes is crucial for developing effective mitigation strategies. These communities experience strong disturbances under antifouling pressure, the implications of which must be considered when developing new technologies due to their potential to alter succession, invertebrate settlement, and invasive species establishment risk. We leveraged the development of a shear-based antifouling technique to examine marine biofilm community assembly and stability under disturbance. The influence of repeated underwater shear on microbial community succession and biofilm matrix stability was assessed over 37 days on uncoated and “foul release” paint-coated surfaces. Foul-release coating decreased matrix biomass, and uncoated and coated surfaces hosted different biofilm communities with converging bacterial compositions and diverging eukaryotic compositions over time. On both surfaces, highly frequent shear strongly shifted the community composition and enriched several shear-tolerant bacteria, diatoms, green algae, and ciliates. Infrequent shear decreased matrix biomass, resulted in weaker compositional shifts and fewer enriched taxa, and additionally prevented macrofouling growth when combined with foul-release coating. A cross-domain co-occurrence network revealed mostly positive correlations persisting through the disturbance continuum and identified the diatom *Melosira* as a highly connected genus. Infrequent shear on anti-biofouling paint-coated surfaces was the most effective biofilm removal strategy, demonstrating that longer recovery periods enabled continued biomass removal and fewer shear-tolerant taxa. The results support the idea that variability in the frequency of a stress disturbance can significantly alter microbial community succession and biomass stability in marine biofilms, resulting in a varied potential for species invasiveness.

**IMPORTANCE:**

Disturbances are major drivers of community succession in many microbial systems; however, relatively little is known about marine biofilm community succession, especially under antifouling disturbance. Antifouling technologies exert strong local disturbances on marine biofilms, and resulting biomass losses can be accompanied by shifts in biofilm community composition and succession. We address this gap in knowledge by bridging microbial ecology with antifouling technology development. We show that disturbance by shear can strongly alter marine biofilm community succession, acting as a selective filter influenced by frequency of exposure. Examining marine biofilm succession patterns with and without shear revealed stable associations between key prokaryotic and eukaryotic taxa, highlighting the importance of cross-domain assessment in future marine biofilm research. Describing how compounded top-down and bottom-up disturbances shape the succession of marine biofilms is valuable for understanding the assembly and stability of these complex microbial communities and predicting species invasiveness.

## INTRODUCTION

Marine bacteria, archaea, and eukaryotes quickly attach to submerged surfaces as biofilms. Biofilms are biogenic habitats because the organisms within generate a communal extracellular polysaccharide (EPS) matrix that alters surface physiochemical properties, enabling nutrient accumulation and facilitating the settlement and growth of larger eukaryotic organisms (1). Biofilms participate in the healthy functioning of ecosystems in environments ranging from intertidal zones to deep marine sediments (2, 3). They host great organismal diversity in close proximity, enabling frequent and dynamic interactions between taxa and a great diversity of functional potential (1, 4). Compared to non-surface-attached communities, communities growing in marine biofilms demonstrate greater resistance against disturbances such as antibiotics, toxic metals, desiccation, ultraviolet (UV) irradiation, shear, and irregular temperature and salinity (5–10). Artificial surfaces represent an abundant and globally distributed habitat for marine biofilm growth. Unlike organismal microbiomes, biofilms on inert artificial surfaces grow independently of host influences, making them a valuable system for studying microbial community dynamics.

Biofilm growth on artificial surfaces—biofouling—is an ongoing challenge to marine operations ranging from transportation and fisheries industries to scientific research (5–10). Biofilm removal or “antifouling” strategies represent artificial disturbances that can facilitate unforeseen effects on the surrounding ecosystem. At the local scale, biofouling removal can seed or alter coastal communities, exert hypoxic pressure on the benthos, and discharge copper or other biocidal chemicals into the environment (11, 12). At the global scale, ships present abundant artificial surfaces that can be hotspots of biofouling growth and may serve as vectors of invasive species transfer (12–14). The primary aim of antifouling technology development has typically been effective biomass removal. However, effects of antifouling disturbance may manifest as responses detectable only in biofilm community composition but not in biomass (15, 16). Especially at microscales, where microbial biofilms may persist despite bulk biomass removal, the effects of the selective pressures applied by antifouling techniques can temporarily or permanently shift community composition, thereby changing succession patterns and potentially enriching tenacious organisms with higher establishment risk, invasiveness, or pathogenicity (17–19). In studying community composition and succession in response to antifouling (20–25), the greatest focus has been on examining the impacts of various surface coatings (19, 26–31). Surface treatments are especially effective in combination with a second antifouling method such as UV light, electrochemical control, ultrasound, or physical abrasion (32–35). When combined, these disturbances exert bottom-up (surface treatment) and top-down (secondary antifouling) controls on biofilms. There has been significant evaluation of the invasiveness risk of baseline biofouling assemblages. However, the implications of antifouling, including compounded disturbances resulting from multiple antifouling approaches on biofilm community structure, have not been broadly considered, despite their potential importance to local ecosystem health and global species transfer.

Surface coatings commonly used on seafaring vessels rely on vessel movement to generate the hydrodynamic shear needed to slough off growth (36). However, necessary biofouling removal during stationary docking periods often relies on abrasive techniques that can damage expensive antifouling coatings, may necessitate biomass capture, and may release coating-associated toxic chemicals (37). As an alternative, another physical antifouling strategy called “grooming” uses frequently repeated and more gentle hull cleaning (11, 38). Mechanical grooming may use gentle abrasion via sponges or brushes (11, 36, 39–41), with grooming schedules adjusted to both minimize surface erosion and maximize biofouling removal. Grooming with contactless shear was recently introduced as a potential sustainable antifouling alternative for ship hulls, using a circular acrylic disk with a water inlet to produce strong wall shear and contactless grip of a surface via radial jet flow (42, 43). In our recent study, image analysis suggested that contactless shear grooming achieved approximately 50% removal of bulk biomass from unpainted surfaces and almost complete removal from painted surfaces (43). Microbial community-scale dynamics of these shear-treated biofilms could provide additional information; biofilm communities can be strongly shaped by shear stress and could potentially respond in ways not seen at bulk biomass level (44). In general, besides influencing the relative level of total biomass removal, biofilm disturbance frequency and intensity may be important in modulating the effects on microbial growth and community composition and diversity (45–47). The effects of technologies used to discourage biofilm growth should therefore be considered at the microbial community level, beyond bulk biomass removal efficiency.

Here, we investigate microbial community dynamics in marine biofilms exposed to foul-release paint and/or shear. We examine the relative impacts of the antifouling-induced disturbance on stability in biomass and bacterial and eukaryotic community composition using samples generated during our prior experiment (43). We consider shear a short “pulse” disturbance and the passive foul-release paint a constant “press” disturbance, both of which potentially alter biofilm stability and recovery. Our null hypothesis was that community succession would be similar irrespective of disturbance type, but any potential enrichment of taxa in response to antifouling treatments was of specific interest. Through network analysis, we identified associations between taxa that point to parallel behaviors of bacterial and eukaryotic taxa persisting under the continuum of disturbance types introduced. Such investigations of marine biofilm community responses to combinations of different disturbance types appear uncommon, despite their potential to cause distinct regime shifts and cascading effects (48). This study explored the responses of marine biofilm biomass and community composition to two types of disturbance separately and in combination. Biofilm stability was investigated from the perspectives of diversity, community composition, individual taxon responses, and taxon correlation networks, which have all been identified as key indicators of community stability in diverse microbial systems (48–50).

## RESULTS

A marine biofilm experiment was conducted in a western North Atlantic coastal system in Narragansett Bay, Rhode Island, USA, in the summer of 2019, using underwater shear and surface coating as antifouling approaches. Biofilms grew *in situ* on unpainted and foul-release-painted plates for 37 days and were groomed at weekly frequencies of 0.5×, 1×, 2×, and 3×. Ungroomed plates were concurrently sampled as controls and are hereafter referred to as “unpainted controls” and “painted controls.” The integrity of the biofilm matrix was assessed by measuring the concentration of transparent exopolymer particles (TEPs). Shifts and co-occurrences of bacterial and eukaryotic communities were assessed between 16 and 37 days post deployment using 16S and 18S rRNA gene amplicon sequencing.

### Responses of the biofilm matrix biomass to foul-release paint (press) and shear-based grooming (pulse)

**FIG 1:**
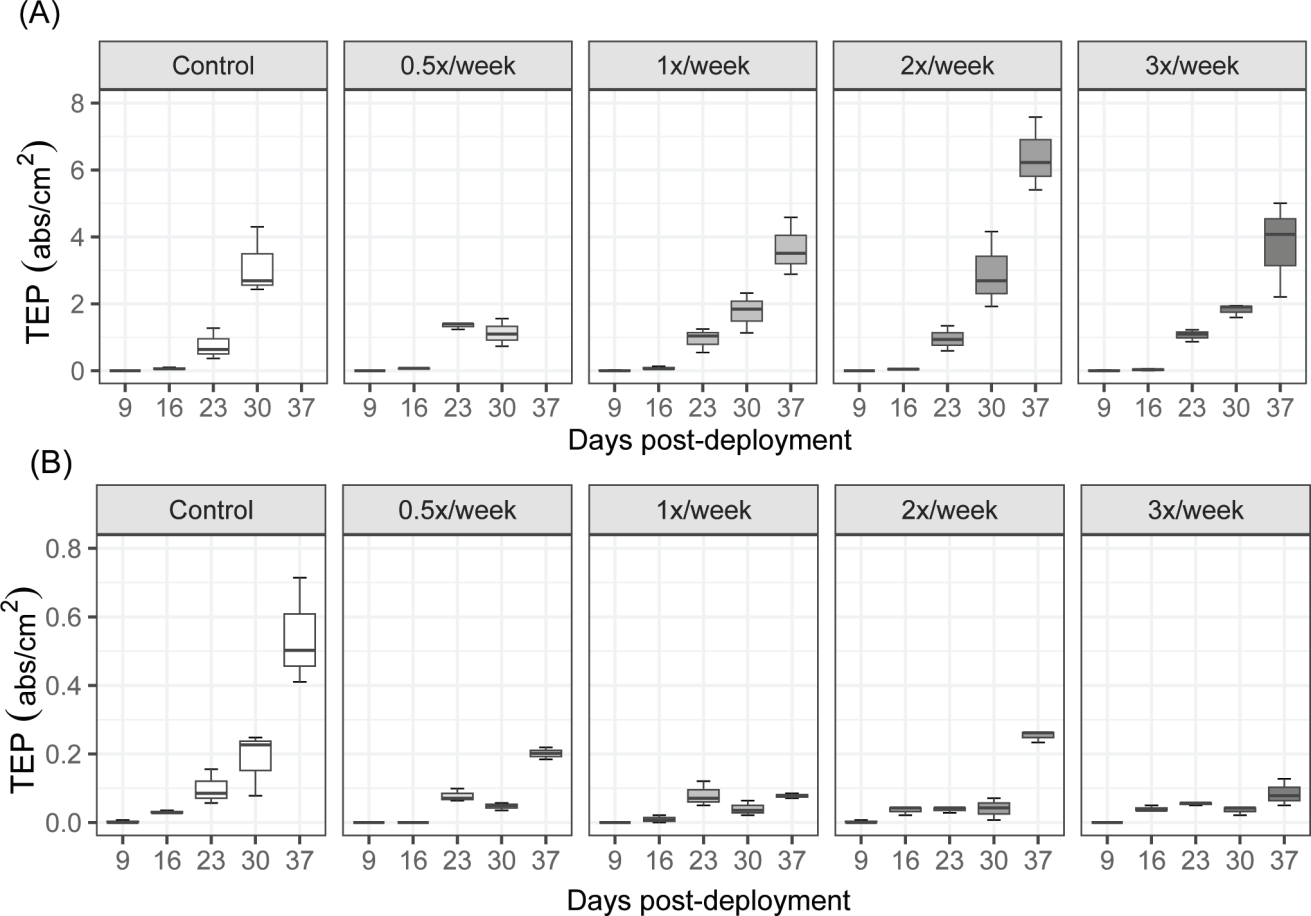
Concentrations of TEPs (absorbance/cm^2^) in biofilms on (**A**) unpainted and (**B**) painted plates until 37 days after deployment. Ungroomed (control) and groomed (0.5×, 1×, 2×, 3×/week) treatments are shown (*n* = 3). Controls and 0.5× samples were not collected from unpainted plates at day 37 due to the growth of calcareous organisms.

TEP was measured between days 9 and 37 post deployment. TEP increased over time on both surfaces and at all grooming frequencies (Fig. 1). Press-only disturbance (painted controls) led to a decrease of TEP concentrations by ~10-fold compared to unpainted controls [repeated measures analysis of variance (rmANOVA); *F* = 14.27, *P* = 0.032, Table 1]; however, biofouling growth still accumulated on painted plates. The influence of pulse disturbance (grooming) was assessed at all frequencies while considering unpainted and painted plates separately. The unpainted controls and pnpainted 0.5× biofilms had similar TEP concentrations until day 23 after which 0.5× biofilms had decreased TEP concentrations compared to controls at day 30 (*t*-test, day 30, *t* = 9.13, Bonferroni *P* < 0.001). Unpainted 1×, 2×, and 3× biofilms had similar TEP concentrations as unpainted controls (Fig. 1A). Considering pulse disturbance on painted plates, grooming resulted in loss of TEP when compared to painted controls in 0.5× (rmANOVA; *F* = 14.27, *P* = 0.032), 1× (rmANOVA; *F* = 35.53, *P* = 0.004), 2× (rmANOVA; *F* = 21.012, *P* = 0.01), and 3× (rmANOVA: *F* = 34.908, *P* = 0.004) biofilms. The combined effect of press and pulse disturbance (groomed painted plates) was then compared to the baseline (unpainted controls) from days 9 to 30. In combination, paint and grooming resulted in significantly lower TEP concentrations at all frequencies (rmANOVAs; *F* > 20, *P* = 0.011 for all frequencies). TEP data were also compared to our previously published chlorophyll *a* data (43) (Fig. S1). When pooling all treatments and controls over time, chlorophyll *a* and TEP concentrations were strongly positively correlated on both unpainted and painted plates (*R*^2^ >0.8, *P* < 0.0001 and *R*^2^ >0.69, *P* < 0.01, respectively).

**TABLE 1:** Summary of the effects of press (surface coating) and pulse (grooming) disturbances on biofilms when considering the entire time course*^a^*

Matrix biomass		**TEP Concentration**									
	Press response	↓									
	***Pulse frequency***	***0.5×***	***1×***	***2×***	***3×***						
	Pulse response	↓	NS	NS	NS						
	Press + pulse response	↓	↓	↓	↓						
											
Bacteria		**Richness**		**Shannon** **Diversity**		**Bray-Curtis**		**Weighted Unifrac**		**Unweighted Unifrac**	
	Press response	↓↑		↓		*		*		*	
	***Pulse frequency***	***0.5×***	***3×***	***0.5×***	***3×***	***0.5×***	***3×***	***0.5×***	***3×***	***0.5×***	***3×***
	Pulse response	NS	↓	NS	↓	*	*	*	*	NS	*
	Press + pulse response	↓	↓	↓	↓	*	*	*	*	*	*
Eukaryotes		**Richness**		**Shannon Diversity**		**Bray-Curtis**		**Weighted Unifrac**		**Unweighted Unifrac**	
	Press response	NS		NS		*		*		*	
	***Pulse frequency***	***0.5×***	***3×***	***0.5×***	***3×***	***0.5×***	***3×***	***0.5×***	***3×***	***0.5×***	***3×***
	Pulse response	NS	↓↑	NS	↓↑	*	*	*	*	NS	*
	Press + pulse response	NS	↓↑	↓↑	NS	*	*	*	*	*	*

^*a*^ The arrows or “*” indicates significant changes in the metric due to disturbance. Downward arrows indicate a decreased value of a metric due to the disturbance, with a consistent effect over the entire period considered, while arrows both up and down indicate different directions of the change at different time points. * indicates different community compositions. “NS” indicates non-significant effects. Disturbance responses in matrix biomass (TEP), alpha diversity, and beta diversity are summarized based on statistical testing performed on the data presented in Fig. 1 to 3, respectively. The results of statistical tests for TEP concentrations and alpha and beta diversity are presented in the Results and in Tables S1-S4, respectively.

### Amplicon sequencing

Microbial community analyses were conducted for samples from unpainted and painted plates collected on days 16, 30, and 37 from controls and from biofilms groomed at the lowest and highest weekly frequencies (0.5× and 3×). In the unfiltered 16S rRNA gene amplicon data set, there were 99,103–170,590 (average 140,351) sequences per sample and 20,202 amplicon sequencing variants (ASVs). After filtering to remove ASVs representing <0.1% of the average sequencing depth and chloroplast and mitochondrial ASVs, there were 56,182–164,724 (average 93,261) sequences per sample and 2,677 ASVs remaining. In the unfiltered 18S rRNA gene data set, there were 58,873–178,449 (average 106,740) sequences per sample and 5,915 ASVs. After <0.1% filtering, there were 56,985–173,716 (average 93,261) and 769 ASVs.

**FIG 2:**
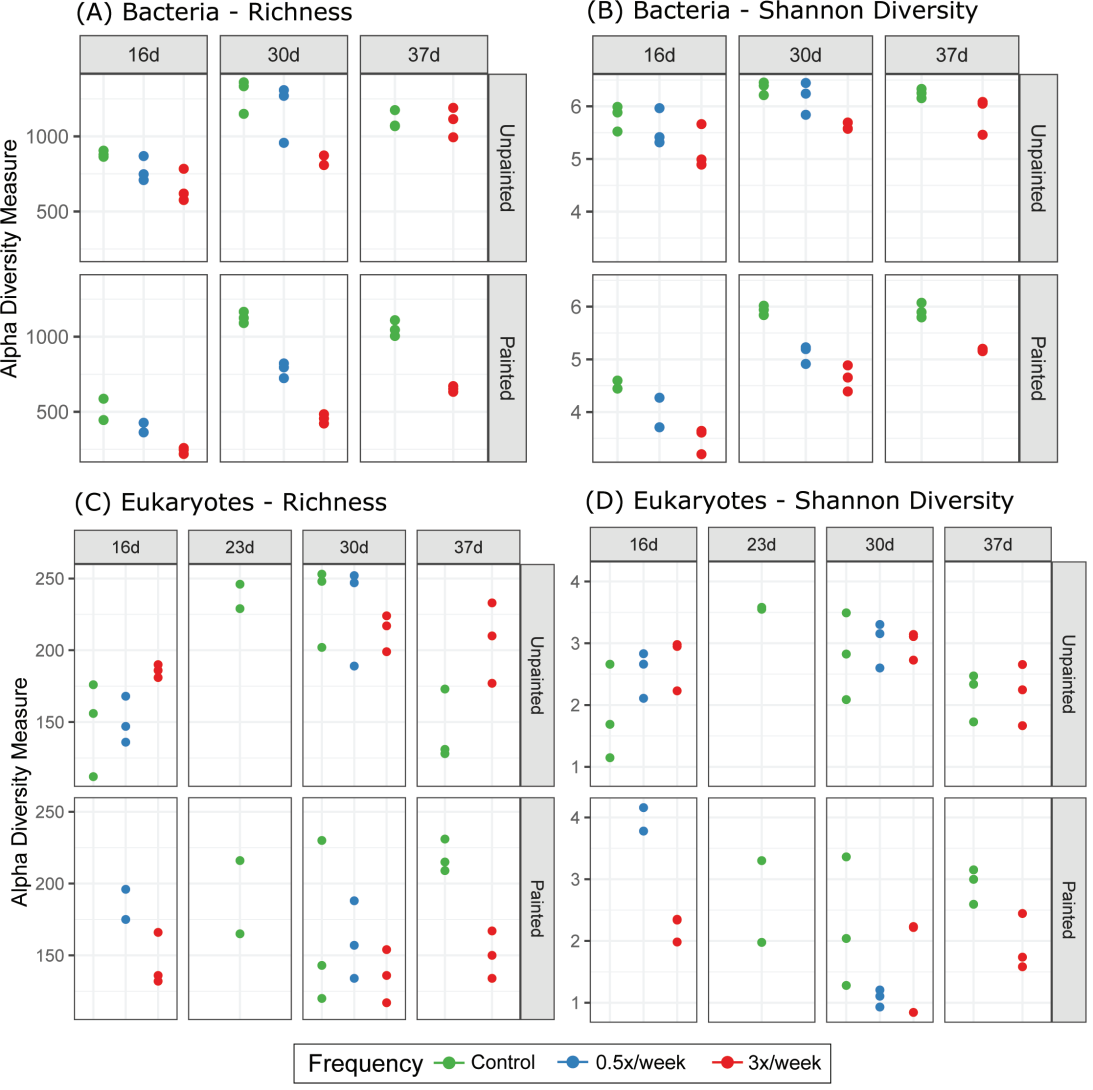
Alpha diversity of biofilm communities. Bacterial (A) richness and (B) Shannon Diversity, and eukaryotic (C) richness and (D) Shannon diversity in control and groomed biofilms at 16, 23, 30, and 37 days.

**FIG 3:**
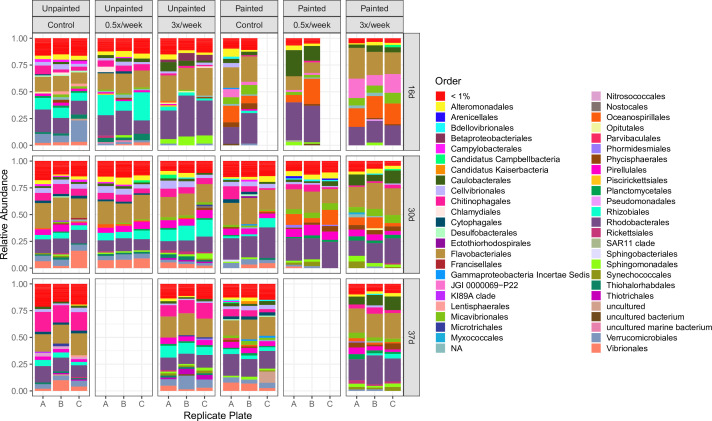
Order-level bacterial community composition in control and groomed biofilms growing on unpainted and painted plates (relative abundance) from 16 to 37 days. (A–C) Replicate experimental plates. Orders that each represented <1% relative abundance in a sample are agglomerated into the <1% category (red).NAs refer to ASVs lacking Order-level classification.

**FIG 4:**
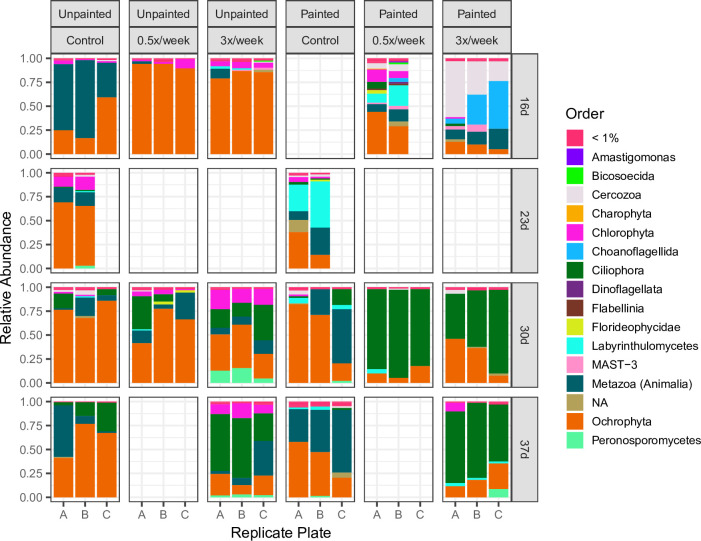
Order-level eukaryotic community composition in control and groomed biofilms growing on unpainted andpainted plates (relative abundance) from 16 to 37 days. Orders that each represented <1% relative abundance in a sample are agglomerated into the <1% category (red).NAs refer to ASVs lacking Order-level classification.

**FIG 5:**
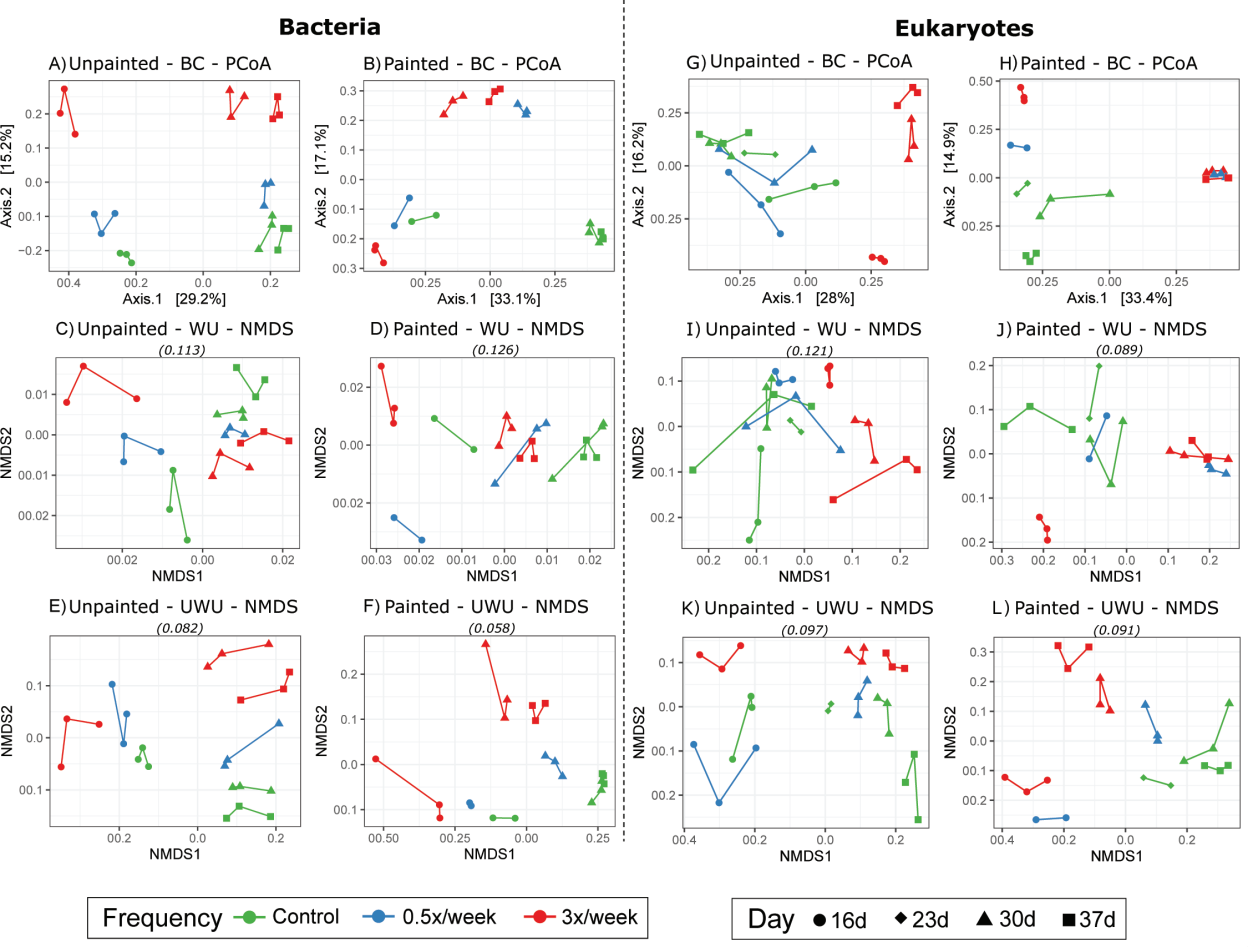
Ordinations representing beta diversity of biofilm communities in control and groomed biofilms up to 37 days after plate deployment. (**A–F**) Bacterial beta diversity. (**G–L**) Eukaryotic beta diversity. BC, Bray-Curtis dissimilarity Principal Coordinate Analysis (PCoA) based on proportions; WU, weighted Unifrac nonmetric multidimensional scaling (NMDS) based on proportions; UWU, unweighted Unifrac NMDS based on rarefied abundances. Stress values for NMDS figures are indicated above each plot in parentheses. Lines connect communities on replicate plates.

Alpha diversity, measured by Richness (observed ASVs) and Shannon diversity, changed over time for bacterial communities (rmANOVA significant “day” effect for controls: Richness *F* = 76.2, *P* < 0.001 and Shannon diversity *F* = 100.9, *P* < 0.001, Table S1), but not for eukaryotic communities (Table S2) (Fig. 2). Compositional changes over time were detectable at the order level (Fig. 3 and 4; Data S1 and 2). Unpainted control biofilm bacterial classes with the highest relative abundances were Bacteroidia (average 29.5%), Alphaproteobacteria (average 25.9%), and Gammaproteobacteria (average 21.26%). Ochrophyta, Metazoa, Chlorophyta, and Ciliophora generally dominated the control eukaryotic communities, while Labyrinthulomycetes temporarily rose to 27%–48% of the community at day 23 on painted plates (Fig. 4). Beta diversity was assessed using Bray-Curtis dissimilarity (BC), Weighted Unifrac (WU), and Unweighted Unifrac (UWU) metrics (Fig. 5). Overall, the effects of sampling day, surface type, and grooming frequency were all significant in permutational multivariate analyses of variance (PERMANOVAs) considering all bacterial samples (Table S3) and all eukaryotic samples (Table S4) for all three beta diversity metrics. Control bacterial and eukaryotic community compositions shifted significantly over time (BC, WU, and UWU: PERMANOVA, significant day effects, Tables S3 and S4).

### Influence of press disturbance (paint) on biofilm bacterial communities

Surface coating with the foul-release paint represented the press disturbance in this study, and its impacts were assessed by comparing unpainted control biofilms to the painted control biofilms over time. There was lower diversity on painted controls than on unpainted controls on day 16, but diversity was similar at days 30 and 37 (Fig. 2). Unpainted and painted controls had distinct bacterial communities in terms of overall relative abundances (BC), taxon occurrence considering phylogenetic distance (UWU), and proportions of taxa considering phylogenetic distance (WU) (Fig. 5, significant “plate type” effect in PERMANOVAs, Table S3). Paint altered bacterial community composition more strongly in 16-day biofilms, with weaker effects in the late 30–37-day biofilms (Fig. S2). At day 16, when compared to unpainted controls, painted controls had lower relative abundances of Chitinophagales, Rhizobiales, Verrucomicrobiales, and Vibrionales and higher relative abundances of the Gracilibacteria JGI 0000069-P22, Micavibrionales, Oceanospirillales, and Rhodobacterales (Fig. 3; Data S1). Paint influenced bacterial community succession (all significant day × surface type interactions, Table S3). Unpainted control and painted control bacterial communities remained distinct throughout the experiment, but community dissimilarities were greatest at day 16 and decreased by days 30–37, indicating a tendency for the community composition to converge on the two surfaces over time (Fig. S2). However, by day 37, compared to unpainted controls, painted controls still had lower proportions of Chitinophagales and higher proportions of Micavibrionales and Sphingomonadales (Fig. 3; Data S1).

### Influence of pulse disturbance (grooming) on biofilm bacterial communities

The influence of pulse disturbance (grooming) was assessed by comparing the biofilms subjected to high-frequency (3×) and low-frequency (0.5×) grooming to ungroomed biofilms (controls) while considering unpainted and painted plates separately.

For unpainted plates, the 3× biofilms were significantly different from unpainted controls in Richness and Shannon diversity (Fig. 2; Table S1). Temporal Richness patterns were different in unpainted control and unpainted 3× biofilms, with Richness increasing in 3× biofilms and plateauing after day 30 in control (significant day × frequency interaction, Table S1). Unpainted control and 0.5× biofilms were similar in terms of both Richness and Shannon diversity, with similar concurrent increases during the shorter 16–30-day period (Fig. 2; Table S1). On the unpainted plates, grooming shifted bacterial community succession and composition overall compared to controls, but these shifts were more strongly associated with more frequent (3×) grooming. Control and 3× biofilms had distinct community succession and composition, which were attributed to both changes in proportions of taxa (WU) and taxon occurrence (UWU) (Table S1; Fig. 5). Unpainted control and 0.5× biofilms had similar patterns of succession, while their compositions were weakly different overall (BC and WU, Table S3).

Considering the effect of grooming on painted plates, 0.5× bacterial communities had weakly lower Shannon diversity, while 3× biofilms had lower Shannon diversity and Richness (Fig. 2; Table S1) than the painted controls. Painted-groomed biofilms had distinct bacterial communities compared to painted controls (Fig. 5; Table S3). The painted 3× biofilm bacterial community composition and succession were strongly different from painted controls in terms of both proportions of taxa (WU) and taxon occurrence (UWU), while these effects were weaker among painted 0.5× biofilms (Fig. 5; Table S3).

**FIG 6:**
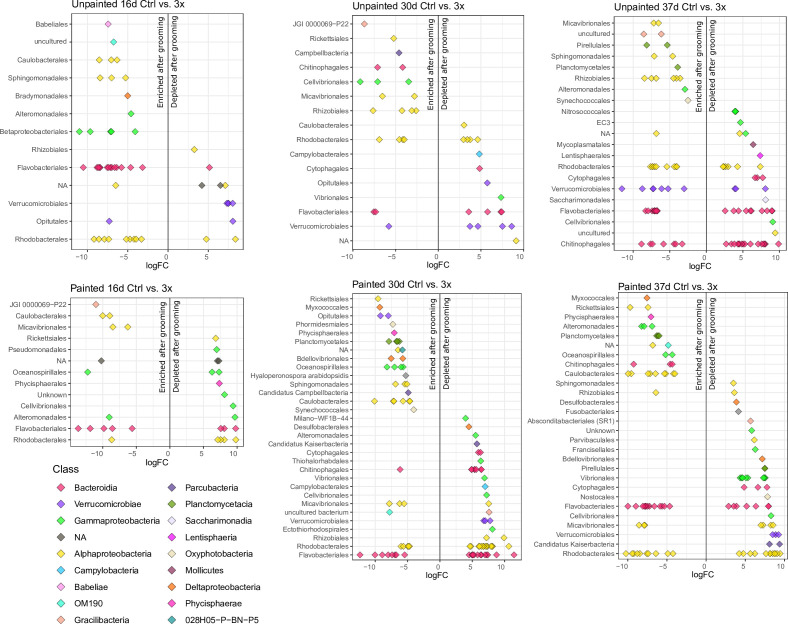
Differentially abundant bacterial ASVs in control and groomed 3× biofilms, measured at 16, 30, and 37 days after deployment on unpainted (top row) and painted (bottom row) plates. More positive and more negative logFC values indicate stronger ASV depletion and enrichment after grooming than the control, respectively. All logFC values were >2 or <−2. Each point represents a single ASV; colors indicate class; and the y-axis represents order-level taxa. NA on the y-axis indicates ASVs missing order-level classification, and the NA ASVs are colored according to class. Further information about enriched and depleted ASVs, including their taxonomy and edgeR statistics, is provided in Data S3.

ASVs that were differentially abundant in control and groomed biofilms were identified using edgeR analysis (Fig. 6). Bacterial ASVs were labeled “enriched” if they had greater abundance in groomed biofilms, and “depleted” if they had greater abundance in control biofilms of the same surface type. On unpainted surfaces, trends in 3× biofilms were first considered due to the stronger compositional shifts. Comparing unpainted plate controls and 3× biofilms at day 16, there were already strong differential patterns among bacterial ASV relative abundances (Fig. 6). At day 30, in 3× biofilms on unpainted plates, only Verrucomicrobiales continued to be generally depleted, while most other trends changed (Fig. 6). By day 37, many orders consisted of several enriched and depleted ASVs at once (Fig. 6). This was especially the case for ASVs in the orders Chitinophagales, Flavobacteriales, and Rhodobacterales. However, orders including Micavibrionales and Rhizobiales continued to be exclusively enriched in these later 3× biofilms. In 0.5× biofilms, few differentially abundant ASVs were detected when compared to unpainted controls (Data S3). At day 16 in 0.5× unpainted plate biofilms, six ASVs (four Flavobacteriales, one Rhodobacterales, and one Alteromonadales) were detected as enriched, and seven ASVs (two Verrucomicrobiales, one Opitulales, one Betaproteobacteriales, and three unclassified ASVs) were detected as depleted after grooming. In 0.5× biofilms on unpainted plates at day 30, only three ASVs showed significance in abundance compared to controls (enriched Flavobacteriales and depleted Campylobacterales and Verrucomicrobiales) (Data S3).

Grooming combined with paint treatment resulted in a greater diversity of taxon responses than paint alone (painted control) (Fig. 6). In painted plate 3× biofilms, the number of differentially abundant ASVs greatly increased, from 31 ASVs at day 16 to 100 and 106 ASVs at days 30 and 37, respectively (Fig. 6; Data S3). Between days 16 and 30, Caulobacterales continued to be enriched in the 3× biofilms. At days 30 and 37, Flavobacteriales and Rhodobacterales ASVs were split between depleted and enriched in 3× biofilms. Most of the order-level trends observed at day 30 continued at day 37 (Fig. 6). By day 37, many more Vibrionales ASVs were depleted in groomed biofilms, while Alteromonadales and Chitinophagales shifted from being mostly depleted at day 30 to being exclusively enriched by day 37.

There were fewer grooming-enriched ASVs detected in 0.5× painted plate biofilms (4 and 22 ASVs at 16 and 30 days, respectively) than in 3× painted plate biofilms (9 and 57 ASVs at 16 and 30 days, respectively). However, enrichment/depletion trends at the order level were very similar to those seen in 3× biofilms (Data S3). Compared to 3× grooming, 0.5× grooming resulted in weaker enrichment of the Gracilibacteria JGI 0000069-P22 at day 16, weaker enrichment of Caulobacterales, and weaker depletion of Campylobacteria at day 30 (Fig. 3).

### Influence of press disturbance (paint) on biofilm eukaryote communities

Paint treatment alone did not change eukaryotic Richness or Shannon diversity compared to unpainted control biofilms (Table S2). Eukaryotic alpha diversity between replicate samples was also much more variable than bacterial diversity (Fig. 2). Paint resulted in a shift in the composition and succession of the biofilm eukaryotic community. Control unpainted and painted plates had different eukaryotic communities in terms of proportions of taxa and taxon occurrence (Table S4, Fig. S3). Eukaryotic community succession also progressed differently on painted and unpainted plates overall and in terms of taxon occurrence (BC and UWU; significant day × surface type interactions, Table S4).

Paint had a greater effect on the eukaryotic community composition of later (30–37 days) biofilms than of 23-day biofilms. We use the 23-day biofilm as a proxy for early eukaryotic growth on painted plates, as 16-day painted eukaryotic communities could not be analyzed due to insufficient DNA yield. Unlike the bacterial communities that appeared to converge, eukaryotic communities on the two surfaces diverged over time (Fig. S3); this divergence was more apparent based on taxon occurrence (UWU; significant day × surface type interaction, Table S4). Visually, at day 23, unpainted plates had higher proportions of Ectocarpales and Ulvophyceae than painted plates, whereas painted plates had higher proportions of the Labyrinthulomycetes *Aplanochytrium*. At day 30, community composition was highly variable among replicated painted plates, with Fragilariales diatoms and the Cnidarian Hydroidolina alternately dominating. By day 37, painted plates had large proportions of metazoans, including nematodes, polychaetes, and copepods, and greater proportions of Bacillariophyceae diatoms, differentiating them from their unpainted counterparts (Data S2).

### Influence of pulse disturbance (grooming) on biofilm eukaryote communities

Effects of grooming on eukaryotic communities were first considered on unpainted plates. No differences in eukaryotic alpha diversity were observed between unpainted control and unpainted 0.5× biofilms. Unpainted 3× biofilms had different Richness and Shannon diversity from unpainted controls (Table S2). Ungroomed and groomed unpainted plate biofilms had distinct eukaryotic communities and different temporal development in terms of both relative abundances of abundant taxa (WU) and taxon occurrence (UWU) (Fig. 5; Table S4). Compared to unpainted controls, unpainted 0.5× biofilms had different eukaryotic communities and temporal development in terms of relative abundances of abundant taxa (WU) but not based on taxon occurrence (UWU), while unpainted 3× biofilms had different communities and temporal development using all metrics (Table S4).

Eukaryotic communities in groomed painted plates were compared to painted plate controls (ungroomed) at days 30 and 37. At days 30–37, painted control and 3× biofilms had different Richness (but not Shannon diversity) over time, but any differences at individual days were not statistically significant. Painted 3× biofilms were different from painted control in terms of both proportions of taxa (WU) and taxon occurrence (UWU) but had similar community succession as painted controls (Table S4; Fig. 5). At day 30, 0.5× painted plate biofilms had similar alpha and beta diversity compared to painted control (Fig. 2 and 5; Tables S2 and S4).

**FIG 7:**
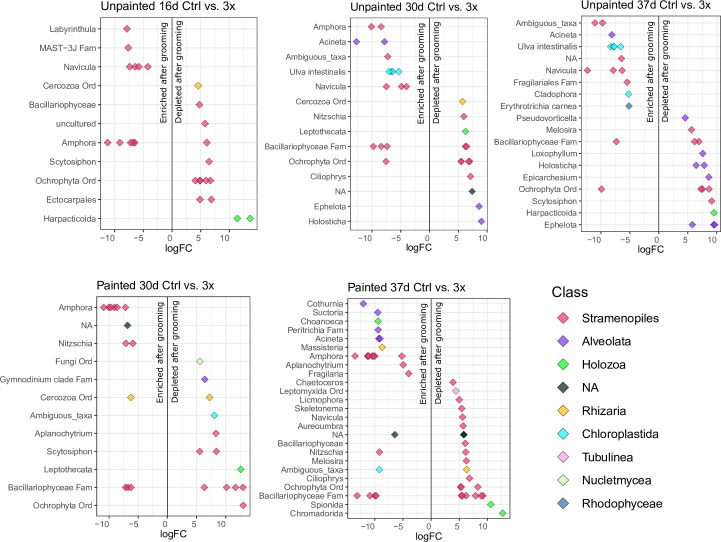
Differentially abundant eukaryotic ASVs in control and groomed 3× biofilms, measured between 16 and 37 days after deployment on unpainted (top row) and painted (bottom row) plates. More positive and more negative logFC values indicate stronger ASV depletion and enrichment after grooming than the control, respectively. All logFC values were >2 or <−2. Each point represents a single ASV; colors indicate class; and the x-axis represents the genus-level taxon information, where available. For ASVs assigned an “uncultured” or NA genus, the genus label indicates order- or family-level classification (e.g., “Cercozoa Ord”), and the NA ASVs are colored according to class. Further information about enriched and depleted ASVs, including their taxonomy and edgeR statistics, is provided in Data S3.

On unpainted plates, notable eukaryotic ASVs enriched in 3× biofilms were associated with the Labyrinthulomycetes genus *Labyrinthula*, the diatoms *Amphora* and *Navicula*, the green algae *Ulva intestinalis* and *Cladophora*, the red alga *Erythrotrichia*, and the ciliate *Acineta* (Fig. 7). Eukaryotic ASVs depleted in 3× biofilms belonged to Cercozoa, brown algae*,* helioflagellates*,* diatoms (including *Melosira*), copepods, and ciliate genera. In 0.5× biofilms, fewer differentially abundant ASVs were detected, with only two ASVs enriched in groomed biofilms (Data S3).

Comparing biofilms on painted control and painted 3× groomed plates, eukaryote ASVs enriched in 3× biofilms again included *Amphora* diatoms. Labyrinthulomycetes (genus *Aplanochytrium*), Cercozoa and *Acineta* ciliates, *Fragilaria* diatoms, a choanoflagellate genus, an unknown Chlorophyceae green algal genus, and several ciliate groups were additionally enriched in the groomed biofilms. ASVs depleted in 3× biofilms were associated with fungi, dinoflagellates, nematode and polychaete genera, helioflagellates*,* and diverse diatoms, including *Melosira*. *Navicula* diatoms were depleted after grooming on painted plates, unlike their consistent enrichment after grooming on unpainted plates. Only four differentially abundant ASVs were identified (three grooming-enriched) on painted 0.5× biofilms (Data S3).

### Influence of press + pulse disturbance on biofilm bacterial and eukaryote communities

The influence of the combination of press and pulse disturbance was assessed by comparing biofilms on painted and groomed plates to those on unpainted and ungroomed plates (unpainted controls). A combination of paint and grooming resulted in strongly different bacterial and eukaryotic communities and different temporal community succession at all grooming frequencies when compared to unpainted and ungroomed plate biofilms (Table 1; Tables S3 and S4).

Bacterial communities were also compared among all biofilms, regardless of surface type and grooming status (Fig. S4). In ordinations representing BC and WU, groomed 30–37-day painted plate biofilms were located near the midpoint of the axis that represents undisturbed community succession, such that they were placed closer to 16-day painted controls compared to equivalent 30- and 37-day controls (Fig. S4A and B). Similar effects of grooming were not observed in painted plate biofilms when considering only taxon occurrence, nor were they observed among unpainted plate biofilms (Fig. S4C). Eukaryotic communities were also compared among all biofilms, regardless of surface type and grooming status. On unpainted plates, 30–37-day 3× biofilms diverged from control and 0.5× biofilms and clustered closer to groomed biofilms from painted plates of similar age (Fig. S5A). This effect was more strongly due to the similar proportions of taxa (WU, Fig. S5B) than to taxon occurrence.

Responses to press (paint) and pulse (grooming) disturbances and combined press and pulse disturbances are summarized in Table 1, including responses in biofilm matrix biomass (TEP concentrations), bacterial and eukaryotic alpha diversity (Richness and Shannon diversity), and bacterial and eukaryotic community composition (three beta diversity metrics).

### Bacteria-eukaryote network through the disturbance continuum

**FIG 8:**
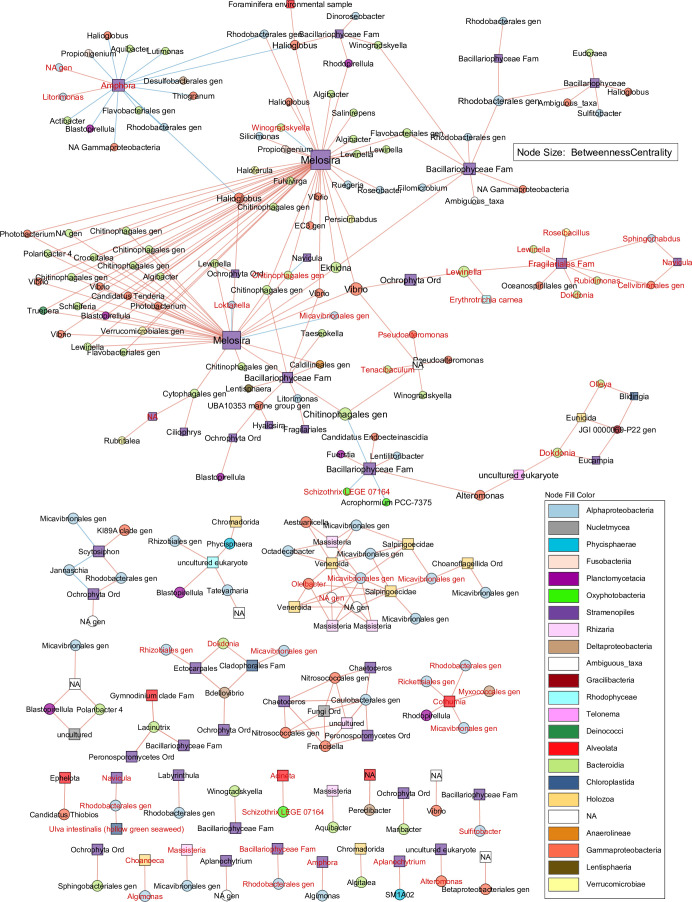
Cross-domain co-occurrence network showing Spearman correlations between bacterial and eukaryotic ASVs. The network represents statistically significant positive (red edges) and negative (blue edges) correlations. Square and round nodes represent eukaryotes and bacteria, respectively. Node shapes and fill colors represent kingdom- and class-level taxonomies. Each node is an ASV, and labels indicate genus. Red labels indicate ASVs that were also identified as shear-tolerant in 3×/week-groomed biofilms using edgeR analysis. When the genus was unknown, the genus label represented the closest identified taxonomic level at either family (e.g., “Rhodobacterales gen”) or order (e.g., “Fungi Ord”). In the larger subnetwork (top half of the figure), node size is positively correlated with betweenness centrality.

To identify stable spatial or ecological associations between the biofilm bacteria and eukaryotes that persisted through the disturbance types in this study, we calculated Spearman correlations between the top 5% bacterial and top 5% eukaryotic ASVs across all treatments and time points. Statistically significant correlations with *r* > 0.8 and *r* < −0.8 were chosen and formed a network with 237 nodes (ASVs) and 273 edges (correlations). The resulting network self-organized into 1 large module, 9 modules with 3 or more nodes, and 19 two-node modules, with mostly positive associations. The network included several ASVs identified as 3×/week shear-tolerant at 16, 30, and 37 days by the edgeR analysis (Fig. 8; Data S4). Key microbial taxa were identified in the largest network module by measuring the betweenness centrality (BT) of the nodes. BT measures how many times a node acts as a bridge along the shortest path between two other nodes (51). In the largest module, the ASVs with the highest BT belonged to the diatom genus *Melosira*. Two *Melosira* ASVs were positively correlated with several individual ASVs and ASV groups. The two *Melosira* nodes were positively but indirectly correlated with each other via a group of Flavobacteriales, Chitinophagales, and Vibrionales ASVs, which in turn had no connections to any other part of the network. Both *Melosira* nodes were connected to an *Amphora* diatom subnetwork via positive correlation with two high-BT *Halioglobus* nodes, which were each negatively correlated with *Amphora*. This shear-tolerant *Amphora* node was positively correlated with two other shear-tolerant Alphaproteobacteria nodes. *Melosira* was directly and indirectly connected to other parts of the network via high-BT Bacillariophyceae/Ochrophyta, Chitinophagales, and *Vibrio* nodes. Other high-BT nodes included several Bacteroidia, diatoms of *Amphora* and the family Fragilariales, and other Bacillariophyceae. Several other shear-tolerant Bacteroidia ASVs were represented throughout this large network module. One “arm” of this network included the majority of shear-tolerant taxa positively associated with Fragilariales and *Navicula* diatoms. The third module was centered around a Rhodophyceae ASV; the fourth represented positive correlations between several Alphaproteobacteria (including some shear-tolerant Micavibrionales) with Venerid mollusc, Choanoflagellate, and Cercozoan nodes. A positively correlated module connected fungi to dinoflagellates and Peronosporomycetes via Gamma- and Alphaproteobacteria. In these smaller modules, Alphaproteobacteria nodes were observed much more frequently and were positively correlated with brown and green macroalgae, ciliates, diatoms, Labyrinthuleans, and unidentified Ochrophyta. A shear-tolerant subnetwork of Alphaproteobacteria was positively associated with a shear-tolerant ciliate, *Cothurnia*. The shear-tolerant groups *Ulva intestinalis* and *Navicula* shared a positive association with the shear-tolerant Rhodobacterales genus. Other notable positive two-node correlations were those between the ciliate *Ephelota* and the *Candidatus Thiobios*, the ciliate *Acineta* and the cyanobacterium *Schizothrix* (both shear-tolerant), and the shear-tolerant Labyrinthulean *Aplanochytrium* and Phycisphaerae.

## DISCUSSION

In this study, we examined shifts in biomass and microbial community responses in growing marine biofilms to individual and combined press- and pulse-disturbance scenarios at a single press intensity, a single pulse intensity, and a varied pulse frequency. Surface treatment via an antifouling paint represented the press disturbance, and shear-based mechanical grooming represented the pulse disturbance. We qualify the paint as a press disturbance because its effect occurred within the recovery period of the grooming-associated pulse disturbance (48). By using multiple beta diversity metrics, we determined that press, pulse, and combined disturbances alter the succession of marine biofilm communities in terms of taxon proportions and their phylogenetic lineages, and that disturbance frequency plays a crucial role in the magnitude of these shifts.

Surface coating by foul-release paint resulted in decreased biomass concentration and altered community diversity and composition, but the observed effects were related to biofilm age and were seen as different successional patterns in bacterial and eukaryotic communities. In the early 16-day biofilm, paint alone did not influence TEP concentrations but maintained lower bacterial diversity and distinct bacterial communities. Chlorophyll *a* data published previously from this experiment showed that paint resulted in only slightly lower 16-day photosynthetic biomass than unpainted (43). Previous studies have reported that foul-release surface coatings do not completely deter initial biofilm formation (16, 27). These effects may be influenced by characteristics of the painted surface, including its color, decreased hardness, and altered wettability (52, 53). Thus, the lower diversity of early bacterial biofilms on painted surfaces compared to unpainted surfaces likely represents a combination of a younger successional stage of painted surface biofilms at 16 days, as well as a community with high attachment potential resulting from selection imposed by bottom-up pressures of the foul-release paint. Diatoms are known to be particularly adherent to silicone-based paints like Intersleek (31). Early dominance of diatoms rather than bacteria may have caused lower initial bacterial diversity, but as we could not identify the early eukaryotic community due to low biomass, this remains to be tested.

In older biofilms, paint-only treatment continued to result in much slower biomass accumulation but affected the biofilm’s bacterial and eukaryotic communities differently. Previously, paint had been shown to greatly decrease photosynthetic biomass between days 30 and 37 (43), and we also show a continuously low, although slightly increasing, TEP concentration until day 30 in this study. While TEP could not be sampled at day 37 due to calcareous fouling accumulation on unpainted plates, it is likely that paint also significantly decreased TEP at day 37 based on the >10-fold decreases at days 23 and 30. However, bacterial and eukaryotic diversity did not change significantly during the same 30–37-day period, and biofilms had similar bacterial diversity on unpainted and painted surfaces. Overall, between 16 and 37 days, bacterial community composition on the two surfaces converged, while eukaryotic communities diverged. Early biofilm communities host dynamic interactions that affect subsequent settlement and growth, and physiochemical characteristics of the environment also influence biofilm succession in different locations and on various surfaces (1, 54). Bacterial communities on six different antifouling coatings have been shown to converge over 94–189 days after immersion, while eukaryotic communities neither converged nor diverged (55). Concurrent bacterial and eukaryotic community convergence has been observed over 56 days in biofilms on nonantifouling surfaces like glass, plastic, and wood, with drivers of bacterial community succession presumably switching from bottom-up effects of the substrate to top-down effects of grazing and predation over time (56). Our findings regarding the convergence of bacterial diversity and community composition over time are in line with these studies, potentially indicating a weakened bottom-up influence in older biofilms. However, divergence in our eukaryotic communities appears to be a new result. On painted surfaces, the oldest biofilms had greater proportions of metazoans such as nematodes, polychaetes, and copepods than unpainted plate biofilms. Larval settlement of select metazoans may be enhanced in the presence of these older biofilms, potentially due to the greater expected variety and concentrations of inductive chemical cues, as observed in *Hydroides elegans*, sponge, and barnacle settlement (57–60). We expect that this late-stage divergence is also partly attributed to the relative enrichment of these mobile predatory and grazing eukaryotes on coated surfaces, which may additionally have contributed to the decreased overall biomass. The convergence of the bacterial community despite eukaryotic community divergence suggests that bacterial introduction via eukaryote settlement may not have played a significant role in biofilm succession. Alternatively, the bacterial associates of new settling eukaryotes may closely resemble already established biofilm bacteria.

We tested the influence of pulse-only disturbance via shear-based grooming of unpainted plates. By day 30, grooming decreased TEP concentration only at the lowest 0.5×/week frequency, while both 0.5× and 3×/week grooming previously showed loss of photosynthetic biomass until day 30 (43). The loss of photosynthetic but not matrix biomass in 3× biofilms may be due to the flexible nature of biofilm EPS, which allows biofilms to behave like viscoelastic fluids at high shear (61). It has been proposed that strong shear can align matrix polymer strands and gather them closer, enhancing electrostatic interactions and hydrogen bonds and resulting in a stronger matrix overall (62). Low-frequency grooming may bypass this strengthening effect due to extended recovery of the matrix between grooming events, thereby allowing matrix removal. However, despite photosynthetic and TEP biomass losses until day 30, 0.5× grooming allowed visible fouling of calcareous organisms to accumulate by day 37, while greater grooming frequencies prevented such growth. The 0.5× grooming also had no effect on bacterial and eukaryotic diversity but did maintain weakly different bacterial and eukaryotic communities. These differences could be traced to fewer than 10 bacterial or eukaryotic ASVs per day. Additionally, when considering only the presence/absence of taxa, 0.5× grooming had no effect on bacterial or eukaryotic communities, suggesting that the effects of grooming are limited to changing the proportions of existing groups rather than replacing them with shear-tolerant counterparts. However, grooming at 3× decreased bacterial diversity and maintained strongly different bacterial and eukaryotic communities, with several apparently shear-tolerant organisms becoming established. These results demonstrate that on unpainted surfaces, shear treatment frequency must be carefully considered due to the tradeoffs between biomass removal and enrichment of shear-tolerant groups. The results suggest that high-frequency shear-based grooming is not recommended without parallel foul-release paint treatment due to the establishment of a shear-tolerant biofilm. Low-frequency grooming is effective, but for a shorter period, and may need to be amended with a secondary antifouling technique to prevent the growth of calcareous organisms. Grooming can therefore extend the “cleanliness” of an unpainted surface, decreasing the frequency at which harsher removal strategies would be needed.

We also assessed the influence of a combined press and pulse disturbance by using the foul-release coating and grooming together. When compared to the baseline of unpainted, ungroomed surfaces, grooming of painted surfaces at all frequencies resulted in significant loss of the photosynthetic biomass (43) and the biofilm TEP matrix. At the two tested frequencies of 0.5× and 3×/week, bacterial communities also demonstrated decreased diversity and richness and distinct compositions according to all metrics used. The eukaryotic communities also had distinct compositions at all frequencies under combined disturbance, but at 0.5× and 3×/week grooming, diversity was similar to that in baseline communities. These results indicate that a combination of foul-release paint and shear-based grooming is an extremely effective antifouling strategy. To further identify an ideal grooming frequency for painted surfaces, we compared groomed and ungroomed biofilms on painted surfaces only. Low-frequency (0.5×/week) grooming of painted surfaces resulted in significant biomass removal and maintained “younger” bacterial communities, despite weaker effects on the biofilm community diversity and composition than high-frequency grooming. However, high-frequency grooming at 3×/week on painted surfaces resulted in strongly different bacterial and eukaryotic communities compared to controls, with a distinct group of shear-tolerant taxa becoming established by day 30. This shows that low-frequency grooming of a biocide-free, foul-release paint-coated surface can effectively remove marine biofilm growth with minimal enrichment of shear-tolerant species and without requiring abrasive removal. The results suggest that high-frequency grooming of the painted surfaces is neither required nor recommended due to the risk of encouraging the growth of extremely shear-tolerant biofilms.

### Components of the shear-tolerant biofilms

High-frequency grooming was associated with the enrichment of several bacterial and eukaryotic groups, forming shear-tolerant biofilms on both unpainted and painted surfaces by the end of the experiment. On unpainted surfaces, most of the shear-tolerant bacterial community changed over time, as seen, for example, in the shift of Flavobacteriales and Rhodobacterales taxa from mostly shear-tolerant in early biofilms to a mix of shear-tolerant and shear-susceptible phyla in older biofilms. These groups are recognized to be abundant in marine biofilms worldwide and likely have significant functional redundancy among their members, which may result in the resilience of community functioning in disturbed biofilms (4, 63). In older biofilms (30–37 days), consistent shear tolerance of Micavibrionales on unpainted plates may have implications for downstream community succession of the biofilms, as they belong to an obligate predatory group of Bdellovibrio and Like Organisms that exert top-down control on communities and whose abundance is positively correlated with microbiome diversity (64, 65). Rhizobiales, also found to be consistently tolerant to frequent shear on unpainted plates, have been previously identified as epibiotic associates of the green algae *Ulva intestinalis* (66). Concurrently, at days 30–37, a shear-resistant, filamentous green alga had become visually abundant on the same plates (43). Our amplicon sequencing evidence indicated that these filamentous green algae were likely *Ulva intestinalis*. Its shear co-resistance with Rhizobiales suggests that this algal-bacterial association may persist in these artificial surface biofilms. *U. intestinalis* enrichment is also likely to have influenced the shear resistance of other bacteria, as filamentous green algae enriched under turbulent flow conditions can modify fluid dynamics around the biofilm, potentially allowing bacterial refuge (67). The diatoms *Amphora* and *Navicula* were also identified as shear-tolerant on unpainted plates; these groups are frequently reported as persistent on antifouling coatings (68–70), but their enrichment under shear on unpainted surfaces further demonstrates their biofouling potential. The ciliate *Acineta* was also tolerant of frequent shear, despite the shear susceptibility of many other ciliate groups. *Acineta* has been previously detected in high proportions in marine biofilms (71). This shear-tolerant set of organisms represents strong attachment potential on unpainted surfaces. Due to the shear-stimulated dominance of potential refuge-providing macroalgae, recovery of the biofilm community to an undisturbed-like state may be limited in the short term after grooming pressure is lifted. However, grazing and invertebrate settlement could eventually allow recovery of a typical biofouling assemblage.

On painted surfaces, high-frequency grooming resulted in a compositionally different shear-tolerant biofilm from those on the unpainted plates; several bacterial groups had become established by day 30 and appeared again at day 37. These included Caulobacterales of the family Hyphomonadaceae, which produce polysaccharide holdfasts that enable strong adherence to smooth surfaces and reportedly thrive on microplastics (72), and Rickettsiales, which are often associated with marine gastropods and molluscs, sometimes as pathogens (73). One consistently shear-tolerant eukaryote on painted plates was the diatom *Amphora*. *Amphora* diatoms can persist under both natural and experimental shear conditions (70, 74, 75). *Amphora* can also survive long-term, brush-based grooming of foul-release surfaces (39) and diatomaceous biofilms that include *Amphora* increase surface friction of foul-release coatings by up to 70% (76). High-frequency grooming during docking could thus promote a hardy *Amphora*-rich biofilm that can increase the surface roughness of the foul-release paint and could survive the lower shear experienced during transport. The other shear-tolerant eukaryotes on painted plates included Labyrinthulomycetes *Aplanochytrium*, cercozoan *Massisteria*, and ciliates such as *Acineta*, which are all potentially bacterivorous (77–79). Macroaggregate association can enable bacterivorous protists to access a boundary layer rich in organic particles and planktonic bacteria (80, 81). Frequent shear may allow these bacterivores to remain in contact with such a boundary layer due to the removal of larger eukaryotes, but further research is required to examine this link. The high-frequency shear used in these experiments allowed us to identify cohorts of shear-tolerant biofilm organisms. The persistence of these organisms may need further consideration when developing antifouling paints and associated antifouling methods.

### Persistent bacteria-eukaryote network in marine biofilms

Disturbance responses are mediated by both individual microbial members and the interactions between them (82). A starting point for identifying these interactions is the construction of co-occurrence networks based on shifting proportions of microbial taxa over time or across disturbances (83, 84). The topology of co-occurrence networks has been used to inform questions regarding keystone species, community stability, and ecosystem functioning in diverse microbial systems (82, 85–90). Marine biofilm network analyses generally focus on bacterial communities (91, 92), but incorporation of eukaryotes into such networks is uncommon despite their abundance in the biofilms (23). The sequencing of both 16S and 18S rRNA gene amplicons from the same biofilm samples enabled the construction of a cross-domain correlation network in this study. Since samples included all treatments and time points, the resulting network represented correlations that persisted across time, surface type, and grooming status and frequency. Most correlations were positive, indicating stable associations between taxa that may involve co-occurrence through succession or grooming-associated co-enrichment and co-depletion. The network modules were divided between a large diatom-centric module and several smaller modules, including pairs of correlated ASVs. Nodes representing the diatom *Melosira* were the most densely positively connected and the most central nodes in the largest module. Grooming depleted *Melosira* in the late biofilms on both surface types, so a portion of its positive bacterial correlations are likely representative of co-removal under shear. *Melosira* has been previously detected in marine biofilms (93–95), including on foul-release coated surfaces (74). These centric diatoms form long filaments and have been reported to dominate arctic sea ice communities as dense, mucilaginous aggregates (96). Production of exopolymeric products by such filaments could have enabled bacterial associations, resulting in a connected community that could have grown and been removed concurrently. Several Bacteroidia were exclusively and positively correlated to *Melosira*; this was unexpected as diatom-Flavobacteriales interactions are commonly reported but not with *Melosira* (97). Several shear-tolerant Bacteroidia were also identified in the network as positively correlated with diatoms and red algae. While the largest network module was diatom-centric and excluded most Alphaproteobacteria, smaller network modules were made of several Alphaproteobacteria that were positively correlated with diatoms, brown and green macroalgae, and many heterotrophic and potentially predatory or parasitic protists, including Cercozoa, Labyrinthulomycetes, Choanoflagellates, and Fungi (77, 78, 98). Among bacteria resistant to protistan digestion, Gamma- and Alphaproteobacteria have shown to be the most abundant, forming stable associations with their grazers potentially due to Type VI and IV secretion systems that facilitate intracellular colonization and suppression of host defenses (99). The frequent Alphaproteobacteria-protist interactions observed in these biofilms likely include such intracellular associations. Another notable positive correlation was between the suctorian ciliate *Ephelota* and Gammaproteobacterium *Candidatus Thiobios*. Plastisphere *Ephelota* has been found to be almost completely covered in an ectosymbiotic, rod-shaped bacteria, similar to how the stalked ciliate *Zoothamnium niveum* is covered in the rod-shaped *Thiobios* (100, 101). Persistent positive correlations under our disturbance continuum lend further evidence to the notion that an ectosymbiotic relationship may exist between *Thiobios* and *Ephelota* in marine biofilms. Another shear-tolerant ciliate, *Cothurnia*, was positively correlated with several shear-tolerant bacteria, which may also indicate bacterial association. The shear-tolerant *Ulva intestinalis* and *Navicula* were also positively correlated via a bacterial node, providing evidence for the potential shear refuge provided by *U. intestinalis*. Taxa similar to the shear-tolerant groups detected in these cross-domain associations have previously been described as being involved in invertebrate settlement. For example, Alphaproteobacteria, including Micavibrionales, Rhizobiales, Rhodobacterales, and Rickettsiales, the Deltaproteobacterium Myxococcales, as well as peritrich and suctorian ciliates, were among microbial taxa that potentially induced coral development under varied reef conditions (102); all of these groups were shear-tolerant in this study and had cross-domain associations in our network analysis. Molecules produced during bacterial quorum sensing can also enhance the settlement of *Ulva* spores (103), while biofilm-dwelling ciliates may induce or deter invertebrate settlement (104, 105). Hence, shear-tolerant biofilms may have altered chemical cue “signatures” that could maintain an altered community overall and influence further settlement during recovery or transplantation. We also identified several correlations whose ecological significance is unknown. However, the persistence of these correlations in biofilms growing on different surfaces and under different shear frequencies urges further study of their roles in marine biofilm stability.

### Conclusions

The use of silicone foul-release coatings is rising globally, and research to increase their efficiency is ongoing. However, they have poor mechanical strength, are prone to tearing and subsequent microplastic shedding (106), and perform poorly in static conditions, requiring dynamic shear conditions for effective biofilm removal (31). We show that infrequent contactless underwater shear-based grooming of foul-release paint-coated surfaces (i) is sufficient for the removal of biofilms accumulated under static conditions and (ii) does not cause significant enrichment of shear-tolerant biofouling organisms. Our results also shed light on the ecology of surface-attached marine microbial communities. Disturbances to the microbial community act as selective filters, either enabling the community to better tolerate future disturbances or knocking out key species or groups, leaving the community more vulnerable (48, 49). Our study reveals that in the case of shear disturbance, the nature of this filter is correlated with shear frequency, with low-frequency shear exerting weaker effects on microbial succession than with high-frequency shear while still achieving significant biomass removal. Frequent (daily) disturbance of reactor biofilms simplified biofilm morphology and community structure and slowed their recovery, while infrequent (weekly) pulses allowed longer recovery intervals that promoted a resilient biofilm morphology and community structure (107). Similarly, in this study, post-grooming recovery was likely a crucial component in allowing continued biomass removal upon grooming and in inhibiting the dominance of shear-tolerant organisms by allowing periodic recolonization and/or regrowth of shear-sensitive taxa.

Frequent shear disturbance of biofilms on coated surfaces strongly altered their communities when compared to uncoated, ungroomed surfaces, pointing to a completely different type of biofilm growth under combined disturbance. Communities in these biofilms overcame both the initial bottom-up selective pressure of the foul-release coating as well as strong, frequent wall shear acting as a top-down control. The strong selective pressure of this combined disturbance and the associated biomass loss into the surrounding environment are likely to affect local assemblages after long-term docking and grooming of coated vessel hulls. Transport of these shear-tolerant biofilms between ports could also be hazardous to the endpoint ecosystem because their strong attachment potential and shear resistance indicate a heightened invasiveness risk. Ardura et al. (2021) used stress resistance to identify potentially invasive taxa carried in ballast water during a trans-equatorial expedition. Their results suggest that planktonic taxa that are (i) widely distributed, (ii) capable of reproducing en route, and (iii) able to withstand environmental stressors like oxygen depletion, warming, darkness, and ammonium spikes pose the greatest transplantation or invasion risk (108). Stress resistance is likely critical in predicting the invasiveness of biofilm taxa, especially since biofilm communities are generally more resistant than their planktonic counterparts (6). Several biofilm taxa are also widely distributed (4), and those that overcame the combined selective pressures of foul-release paint and frequent, strong shear are prime candidates for further examination of their invasiveness potential. Several shear-tolerant taxa in our cross-domain interactions may also be involved in inducing and deterring macroalgal and invertebrate settlement. If successfully transplanted, the newly introduced mix of chemical cues from these communities could have unintended consequences for the settlement or metamorphosis of local organisms.

The findings in this study raise several additional questions, such as the role of macroalgal refuge in shear tolerance and the mechanisms by which shear-tolerant biofilms recover post-disturbance. Macroalgal refuge and other biotic interactions may continue to shape the biofilm community under disturbance, creating a feedback loop that modifies the effect of the disturbance. Accounting for this biotic feedback will be essential when considering biofouling management. Further study of post-disturbance recovery in a shear-tolerant biofilm (especially on coated surfaces) could test whether this alternative community state, produced by strong and frequent disturbances, is stable or transient.

## MATERIALS AND METHODS

### Experimental setup

From June to July 2019, biofilms were grown on test plates in the Stillwater basin of Narragansett Bay, Rhode Island, USA (41°32′06.8″ N and 71°18′49.8″ W) along a pier of the Naval Undersea Warfare Center’s Test Facility (43). The following two types of 10.2 × 10.2 cm plates were deployed: inert, fiberglass-epoxy surfaces (Garolite G10, McMaster Carr, Elmhurst, IL, USA) (unpainted), and plates of the same material coated on one side with the commercial foul-release Intersleek 1100 SR paint (painted). Plates were spray-painted according to paint manufacturer instructions at the Florida Institute of Technology’s Center for Corrosion and Biofouling Control. Foul-release paint was considered a press disturbance because the paint is designed to disrupt natural biofilm formation and exerts this disturbance continuously throughout the experimental duration (109). Plates were attached to a frame made of PVC pipe and stainless steel; in total, 10 frames with 4 plates each were deployed, with sets of unpainted and painted plates alternated along the pier. The top of the submerged frame was secured by a pair of nylon ropes and remained at least 0.3 m below the lowest tide throughout the experiment. To address potential shading bias from pilings and other structures, the plate sets were rotated three times a week by moving them one position at a time along the pier. Plate submersion, encompassing both grooming and sampling, lasted 37 days.

### Shear-based mechanical grooming

Shear-based biofilm removal was carried out repeatedly using a Bernoulli pad device, which is a circular, clear acrylic disk with an inlet at its center, through which an attached pipe pumps seawater (42). When in proximity to a submerged parallel surface, the jet of seawater produces a radial outflow between the pad and the surface. At an equilibrium gap height, the radial outflow holds the plate surface in a contactless grip. The combination of wall shear and contactless grip is used here as a non-abrasive, underwater antifouling approach. This antifouling approach was considered a series of pulse disturbances, as they were discrete disturbance events with short individual durations (109). Of the 40 deployed plates, 16 unpainted and 16 painted plates were groomed in sets of 4 using contactless underwater shear at four weekly frequencies (grooming approach): once every 2 weeks (“0.5×”), 1×/week (“1×”), 2×/week (“2×”), and 3×/week (“3×”) for 37 days. To groom plates, a single frame with four plates was removed from the pier location at a time and then transferred indoors into a holding tub containing fresh *in situ* seawater within 30 s of removal. The Bernoulli pad was used to groom plates in an indoor tank filled with seawater from the same site. A water pump submerged in the grooming tank pulled the tank’s seawater through a bonded filter and then pushed it downward in a water jet through a submerged Bernoulli pad. The removed biofouling material did not re-enter the water jet. A set of four plates were set flush into an immersed, neutrally buoyant Garolite G10 “grooming frame” (43), then submerged into the grooming tank, and brought upward into the water jet (perpendicular to the water jet and parallel to the Bernoulli pad) until contactless grip and equilibrium were achieved. Plates were moved laterally until visible fouling removal stopped, as gaged through the transparent Bernoulli pad, lasting approximately 1 min. The remaining eight plates (four unpainted and four painted) were not groomed and served as controls. All plates remained immersed in filtered seawater from the deployment site except when transferring between frames, and the work was conducted in a space next to the deployment site; therefore, exposure to air and direct sunlight was minimal. Descriptions of the computational fluid dynamics simulations informing the dimensions of the Bernoulli pad and further discussions of the fluid power, flow rate, wall shear stress, and experimental setup used in this study have been published previously (42, 43).

### Sample collection

Biofilm samples were collected once a week on the same day of the week at 9, 16, 23, 30, and 37 days after plates were immersed. Samples were collected from three replicate plates each of the unpainted and painted control and groomed plate sets treated with shear-based mechanical grooming at four different weekly frequencies. The fourth plate in each set was used for photography and image analyses described previously (43). For sampling biofilms, a 3-D printed sampling device was affixed to a plate, forming 16-mm leak-proof wells (71). Two wells were sampled each time, and each plate area was only sampled once. One milliliter of 0.2 µm filtered seawater was added to the well; the biofilm was completely scraped off with a sterile plastic pestle; and the resuspended biofilm was transferred to sterile microcentrifuge tubes. Biofilm from one well was used for biomass analyses, including chlorophyll *a* (43) and TEP, and the biofilm from the second well was used for DNA-based analyses. DNA sample tubes were centrifuged; the supernatant was discarded; and the biofilm pellets were stored at −20°C on site for the day. The TEP samples were maintained at ~23°C under ambient light and processed the next day. Samples reserved for DNA-based analyses were stored at −80°C until DNA extraction. Only one plate was sampled at a time, while the remaining plates remained submerged in an indoor tank containing filtered seawater from the deployment site.

### Transparent exopolymer particles

TEP concentrations were obtained using the method modified from Passow and Alldredge (110, 111) and previously used to measure marine biofilm TEP (71). Briefly, biofilm suspensions were vacuum-filtered, rinsed, and dried onto 0.2 µm track-etched polycarbonate filters (GVS). Filters were stained with an Alcian Blue solution; stained filters were incubated in 80% H_2_SO_4_; and absorbance was measured at 787 nm using a Fisher Unico 1000 spectrophotometer. Using SPSS v28 (IBM Corp. 2019), differences in TEP concentrations among treatments were tested using two-way rmANOVA comparing the two plate types and four grooming frequencies vs control. Sphericity was assessed using Mauchly’s test (112), and when data violated the sphericity assumption, *P*-values were generated by the Greenhouse-Geisser correction (113). TEP concentrations were compared with chlorophyll *a* concentrations previously published (43) using rmANOVA.

### DNA extraction and PCR amplification

DNA samples from control plates and from biofilms groomed at the lowest and highest grooming frequencies were included in the community analysis. Control and 3× biofilms were analyzed from days 16, 30, and 37, and in 0.5× biofilms from days 16 and 30. The 3× biofilms had been groomed 6, 12, and 15 times by days 16, 30, and 37, respectively. The 0.5× biofilms had been groomed once by day 16 and twice by day 30. These two grooming frequencies were chosen for further analysis as sampling consistently occurred 1 day after grooming for both treatments. DNA was extracted from biofilm pellets following a phenol-chloroform protocol modified from a previous study (114). Briefly, biofilm pellets were resuspended in extraction buffer and 10% sodium dodecyl sulfate (SDS) and transferred to sterile bead beater tubes containing a mixture of 0.1 and 0.5 mm glass beads (Biospec, Bartlesville, OK, USA), followed by agitation for 2.5 min in a Mini Beadbeater (Biospec, OK) and incubation with 10% lysozyme and 20 mg mL^−1^ proteinase K for 30 min at 37°C. DNA was purified from the lysate by extraction with phenol:chloroform:isoamyl alcohol (25:24:1) and chloroform:isoamyl alcohol (24:1). DNA was precipitated overnight with a 0.6× volume of 100% isopropanol (~360 µL), rinsed 2× with 500 µL of 70% ethanol, eluted with nuclease-free water to a final volume of 100 µL, and stored at −80°C until PCR amplification. Bacterial and eukaryotic community compositions in the biofilms were determined via 16S rRNA and 18S rRNA gene amplicon sequencing, respectively. DNA concentrations were measured by PicoGreen, and extract concentrations and PCR template volumes were normalized to ~2 ng DNA per PCR reaction. PCR was conducted with duplicate reactions per sample and primer pair. The hypervariable V4 region of the 16S rRNA gene was amplified with the 341F and 785R primers using a nested PCR approach to minimize amplification biases (115). The V8–V9 region of the 18S rRNA gene was amplified using the V8F (116) and EukBR (117) primers as described previously (71). Following purification using the AxyPrep Mag protocol (Axygen, Union City, CA, USA), the amplicons were barcoded with Nextera dual indexes (Illumina, San Diego, CA, USA) for 8 cycles, purified again, quantified using the PicoGreen dsDNA assay, and adjusted to 6 nM (118, 119). One negative control was processed during both the bacterial and eukaryotic library preparations. A multiplexed library of 96 samples was sequenced at Tufts University using the Illumina MiSeq platform (2× 300 PE).

### Sequence analysis

Sequences were demultiplexed at the sequencing facility and then analyzed using QIIME2 (v.2021.2) on the Massachusetts Green High Performance Computing Cluster (120). Primer sequences and their reverse complements were trimmed using the cutadapt v3.2 plugin for QIIME2 (121), and untrimmed sequences were discarded. For bacterial samples, the forward and reverse reads were truncated according to read quality at base positions 251 and 197, respectively. For eukaryotic samples, forward and reverse reads were truncated at base positions 210 and 163, respectively. Using DADA2, sequences were paired, denoised, dereplicated, and subjected to chimera removal with a --p-min-fold-parent-over-abundance threshold of 1 (122). Following this step, no sequences remained in the negative control samples. Taxonomy was assigned to ASVs with a naive Bayes classifier trained on sequences from the SILVA v132 database at 99% sequence similarity using only the regions of the 16S and 18S rRNA genes targeted by our PCR primers (123, 124). From both bacterial and eukaryotic samples, ASVs whose counts represented <0.1% of the mean sequencing depth were removed (125). From the bacterial samples, chloroplast and mitochondrial ASVs were additionally removed. Phylogenetic trees were created from similarly filtered representative sequences in QIIME2 using the “Phylogeny Align-To-Tree-Mafft-Fasttree” method, which roots trees at the midpoint. Multichotomies in these trees were resolved using the multi2di function from the ape R package (126). All subsequent data analyses were conducted with this filtered data set, and any additional data filtering is noted explicitly. Due to insufficient DNA yield for PCR amplification, eukaryotic communities on 16-day control biofilms on painted plates could not be analyzed, so control biofilm communities at days 23, 30, and 37 were considered in comparisons. Analyses and plotting were performed in RStudio (R 4.2.1) using the Phyloseq package (v1.40.0) (127–129). Alpha diversity was estimated from filtered data using the Richness and Shannon index calculated in Phyloseq and statistically compared with rmANOVAs after testing for data normality and sphericity using IBM SPSS Statistics v28.

Beta diversity at the ASV level was assessed using three different metrics to understand different sources of community shifts. We used the Bray-Curtis metric to quantify dissimilarity in terms of relative abundance without phylogenetic consideration. We used the Weighted Unifrac metric to quantify dissimilarity when considering the phylogenetic distance of taxa, with greater sensitivity to changes in the relative abundance of abundant taxa. We also used the Unweighted Unifrac metric, which is more sensitive to changes across all taxa regardless of abundance, to determine taxon presence/absence via the unshared fractions of total phylogenetic diversity between communities (“taxon occurrence”). Rooted phylogenetic trees created in QIIME2 were used to create non-metric multidimensional scaling ordinations based on Unweighted and Weighted Unifrac distances. Unweighted Unifrac distances were calculated from ASV count data that were rarefied to a minimum sequencing depth of 56,182, while Weighted UNIFRAC distances were calculated using ASV relative abundances (130). Principal Coordinate Analysis ordinations were also generated from ASV relative abundances based on Bray-Curtis dissimilarity matrices. Using the vegan (v2.6-2) package for R, data dispersion was tested with betadisper, and statistically significant differences between community compositions were examined using PERMANOVA with adonis2 (131).

### Identification of differentially abundant ASVs

Differentially abundant ASVs in various treatment groups were identified using edgeR in Phyloseq (132). Data sets were first divided into subsets, including one time point and one surface type per set. Each data subset underwent additional filtering that removed ASVs with low read abundance variance (<10^−6^ across samples) and ASVs present in one or no samples per subset (133, 134). Next, these filtered data sets were converted to Differential Gene Expression data objects for the edgeR package and normalized using the Relative Log Expression method (132, 133). Each edgeR comparison consisted of pairwise exact *t*-tests between control, 0.5×/week-groomed, and 3×/week-groomed communities on the same surface type from a single sampling time point. Statistically significant ASVs (*P* < 0.05) with low false discovery rates (*P* < 0.01) were considered differentially abundant. Relative abundance and ASV differential abundance data were plotted using the ggplot2 and Polychrome packages in R (135, 136).

### Cross-domain network analysis

Taxon co-occurrences were determined between ASVs derived from 16S and 18S rRNA gene amplicon analyses using the Sparse Correlation Network Investigation for Compositional Data (SCNIC) workflow (137). To uncover robust co-occurrences, we included all biofilm samples with both bacterial and eukaryotic community data, including control biofilms and biofilms groomed at both 0.5×/week and 3×/week frequencies from both unpainted and painted plates. For this analysis, only samples with both 16S and 18S data were included, which resulted in 88 of 94 total samples being used for the analysis (44 + 44). To uncover robust associations, only the top 5% of ASVs from the 16S data set (925 ASVs excluding chloroplast and mitochondrial ASVs) and from the 18S data set (296 ASVs) were separately included in the network; these ASVs represented a subset of the ASVs included in all other analyses. Positive and negative Spearman correlations with Benjamini-Hochberg-adjusted *P*-values of <0.05 (to correct for Type I error) were selected for visualization. Network visualization, filtering, and analysis were performed in Cytoscape v3.9.1 (138).

## Data Availability

Sequence reads from 16S and 18S rRNA gene amplicon sequencing are available with NCBI accession number PRJNA888416.

## SUPPLEMENTAL MATERIAL

Data S1 msphere.00248-23-s0001.html Relative abundances of bacterial orders: interactable HTML file with order names and relative abundances (proportions from 0 to 1 per sample).

Data S2 msphere.00248-23-s0002.html Relative abundances of eukaryotic orders: interactable HTML file with order names and relative abundances (proportions from 0 to 1 per sample).

Data S3 msphere.00248-23-s0003.xlsx Results of edgeR differential abundance analysis.

Data S4 msphere.00248-23-s0004.xlsx Network node information.

Supplemental Tables and Figures msphere.00248-23-s0005.docx Tables S1-S4; Figures S1-S5.

ASM does not own the copyrights to Supplemental Material that may be linked to, or accessed through, an article. The authors have granted ASM a non-exclusive, world-wide license to publish the Supplemental Material files. Please contact the corresponding author directly for reuse.
