# Supplementary material for: Disturbance frequency directs microbial community succession in marine biofilms exposed to shear
Source: mSphere. 2023 Oct 16;8(6):e00248-23. doi: 10.1128/msphere.00248-23 (PMC10790581; doi:10.1128/msphere.00248-23)
Supplement: Supplemental Tables and Figures — Tables S1-S4; Figures S1-S5. [file msphere.00248-23-s0005.docx]

**Supplementary Tables and Figures**

**Disturbance frequency directs microbial community succession in marine biofilms exposed to shear**

Abhishek T. Naik^a,b^, Kristina M. Kamensky^c^, Aren M. Hellum^c^, Pia H. Moisander^a,b^#

^a^Department of Biology, University of Massachusetts Dartmouth, North Dartmouth. MA, USA

^b^School of Marine Science and Technology, University of Massachusetts Dartmouth, New Bedford, MA, USA

^c^Naval Undersea Warfare Center, Newport, RI, USA

Running Title: Marine biofilm succession under shear disturbance

#Corresponding author: Pia H. Moisander, pmoisander@umassd.edu

**Table S1**. Repeated measures ANOVA results for bacterial alpha diversity. Comparisons were calculated among filtered abundance data. Day and Day*Type and Day*Freq are within-subject effects, and Freq is a between-subjects effect.

| **Disturbance** | **Effects tested** |  | **Richness** | | **Shannon** | |
| --- | --- | --- | --- | --- | --- | --- |
|  |  |  | **F** | **p** | **F** | **p** |
| **Press** | **Controls: Effect of surface type** (UP controls and P controls; 16, 30, 37d) | **Day** | 76.203 | <0.001 *** | 100.907 | <0.001 *** |
|  |  | **Day*Type** | 6.201 | 0.035 * | 24.396 | 0.001 *** |
|  |  | **Type** | 19.208 | 0.022 * | 41.609 | 0.008 ** |
| **Pulse** | **Unpainted: Effects of 0.5X Grooming** (UP Controls and UP 0.5X; 16, 30d) | **Day** | 42.722 | 0.003 *** | 80.862 | <0.001 *** |
|  |  | **Day*Freq** | 0.001 | 0.976 | 0.18 | 0.693 |
|  |  | **Freq** | 1.889 | 0.241 | 0.947 | 0.386 |
|  | **Unpainted: Effects of 3X Grooming** (UP Controls, UP 3X; 16, 30, 37d) | **Day** | 34.947 | <0.001 *** | 12.693 | 0.003 *** |
|  |  | **Day*Freq** | 11.92 | 0.004 *** | 1.103 | 0.377 |
|  |  | **Freq** | 24.458 | 0.008 ** | 14.511 | 0.019 * |
|  | **Painted: Effects of 0.5X Grooming** (P Controls and P 0.5X; 16, 30d) | **Day** | 710.401 | 0.001 *** | 86.778 | 0.011 * |
|  |  | **Day*Freq** | 26.565 | 0.036 * | 0.683 | 0.495 |
|  |  | **Freq** | 14.064 | 0.064 | 18.721 | 0.049 * |
|  | **Painted: Effects of 3X Grooming** (P Controls and P 3X; 16, 30, 37d) | **Day** | 160.978 | <0.001 *** | 77.37 | <0.001 *** |
|  |  | **Day*Freq** | 27.378 | <0.001 *** | 1.895 | 0.23 |
|  |  | **Freq** | 363.178 | <0.001 *** | 1116.988 | <0.001 *** |
| **Press+ Pulse** | **Combined effect of Paint and 0.5X Grooming** (UP Controls, P 0.5X; 16, 30d) | **Day** | 87.61 | 0.003 *** | 67.482 | 0.004 *** |
|  |  | **Day*Freq** | 0.03 | 0.874 | 9.517 | 0.054 |
|  |  | **Freq** | 104.356 | 0.002 *** | 69.457 | 0.004 *** |
|  | **Combined effect of Paint and 0.5X Grooming** (UP Controls, P 3X; 16, 30, 37d) | **Day** | 77.166 | <0.001 *** | 51.321 | <0.001 *** |
|  |  | **Day*Freq** | 21.005 | <0.001 *** | 15.522 | <0.001 *** |
|  |  | **Freq** | 413.647 | <0.001 *** | 460.717 | <0.001 *** |

**Table S2**. Repeated measures ANOVA results for eukaryotic alpha diversity. A one-way ANOVA was used for Painted Controls vs. 0.5X due to a single time point being available. Comparisons were calculated among filtered abundance data. Day and Day*Type and Day*Freq are within-subject effects, and Freq is a between-subjects effect.

| **Disturbance** | **Effects tested** |  | **Richness** | | **Shannon** | |
| --- | --- | --- | --- | --- | --- | --- |
|  |  |  | **F** | **p** | **F** | **p** |
| **Press** | **Controls: Effect of surface type over time** (UP controls and P control; 23, 30, 37d) | **Day** | 4.788 | 0.087 | 2.412 | 0.205 |
|  |  | **Day*Type** | 11.055 | 0.023 * | 5.633 | 0.069 |
|  |  | **Type** | 0.184 | 0.71 | 0.197 | 0.701 |
| **Pulse** | **Unpainted: Effects of 0.5X Grooming** (UP Controls and UP 0.5X; 16, 30d) | **Day** | 21.658 | 0.01 ** | 2.922 | 0.163 |
|  |  | **Day*Freq** | 0.043 | 0.847 | 0.323 | 0.6 |
|  |  | **Freq** | 0.007 | 0.936 | 4.624 | 0.098 |
|  | **Unpainted: Effects of 3X Grooming** (UP Controls, UP 3X; 16, 30, 37d) | **Day** | 7.359 | 0.015 * | 2.29 | 0.164 |
|  |  | **Day*Freq** | 3.589 | 0.077 | 0.814 | 0.477 |
|  |  | **Freq** | 16.115 | 0.016 * | 8.451 | 0.044 * |
|  | **Painted: Effects of 0.5X Grooming** (P Controls and P 0.5X; 30d)* | **Freq** | 0.016 | 0.906 | 3.492 | 0.135 |
|  | **Painted: Effects of 3X Grooming** (P Controls and P 3X; 30, 37d) | **Day** | 2.555 | 0.185 | 0.968 | 0.381 |
|  |  | **Day*Freq** | 0.838 | 0.412 | 0.382 | 0.57 |
|  |  | **Freq** | 10.443 | 0.032 * | 3.391 | 0.139 |
| **Press+ Pulse** | **Combined effect of Paint and 0.5X Grooming** (UP Controls, P 0.5X; 16, 30d) | **Day** | 2.035 | 0.249 | 3.115 | 0.176 |
|  |  | **Day*Freq** | 6.537 | 0.083 | 12.524 | 0.038 * |
|  |  | **Freq** | 1.144 | 0.363 | 2.426 | 0.217 |
|  | **Combined effect of Paint and 0.5X Grooming** (UP Controls, P 3X; 16, 30, 37d) | **Day** | 4.286 | 0.054 | 0.248 | 0.786 |
|  |  | **Day*Freq** | 7.397 | 0.015 * | 1.584 | 0.263 |
|  |  | **Freq** | 12.649 | 0.024 * | 4.268 | 0.108 |

**^a^**One-way ANOVA

**Table S3**. PERMANOVA results for bacterial beta diversity. Bray-Curtis and Weighted Unifrac dissimilarities were calculated based on filtered proportion data. Unweighted Unifrac distances were calculated based on filtered rarefied (abundance) data. Results in italics are from data subsets with non-homogenous variances according to a betadisper test.

| **Dist- urbance** | **Effects tested** |  | **Bray-Curtis** | | **Weighted Unifrac** | | **Unweighted Unifrac** | |
| --- | --- | --- | --- | --- | --- | --- | --- | --- |
|  |  |  | **F** | **Pr(>F)** | **F** | **Pr(>F)** | **F** | **Pr(>F)** |
| **All** | **All Samples: Effects of surface type, grooming** (UP controls, UP groomed, P controls and P Groomed, all freq: 16, 30, 37d; permutations constrained by plate number) | **Day** | 11.2871 | 0.001*** | *14.1244* | *0.001**** | 15.3547 | 0.001*** |
|  |  | **Type** | *24.7189* | *0.001**** | 28.0486 | 0.001*** | *21.2817* | *0.001**** |
|  |  | **Freq** | *8.2134* | *0.001**** | 8.3596 | 0.001*** | *9.2005* | *0.001**** |
|  |  | **Day*Type** | 6.8902 | 0.001*** | 6.9386 | 0.001*** | 4.6469 | 0.003*** |
|  |  | **Day*Freq** | 3.3347 | 0.006*** | 4.4487 | 0.001*** | 3.0051 | 0.008*** |
|  |  | **Type*Freq** | 3.9845 | 0.001*** | 3.743 | 0.001*** | 2.9303 | 0.001*** |
|  |  | **Day*Type***  **Freq** | 3.3797 | 0.003*** | 4.3666 | 0.001*** | 2.1903 | 0.067 |
| **Press** | **Controls: Effect of surface type** (UP controls and P control; 16, 30, 37d) | **Day** | 6.2307 | 0.001*** | 5.2276 | 0.001*** | 8.3596 | 0.001*** |
|  |  | **Type** | 6.9867 | 0.001*** | 7.0161 | 0.001*** | 6.3691 | 0.001*** |
|  |  | **Day*Type** | 4.0834 | 0.001*** | 3.8125 | 0.001*** | 2.5231 | 0.008** |
| **Pulse** | **Unpainted: Effects of all Grooming** (UP Controls, UP 0.5X and UP 3X; 16, 30, 37d) | **Day** | 11.2537 | 0.001*** | 13.851 | 0.001 *** | 10.809 | 0.001 *** |
|  |  | **Freq** | 6.1159 | 0.001*** | 5.036 | 0.001 *** | 4.5008 | 0.001 *** |
|  |  | **Day*Freq** | 3.8914 | 0.001*** | 4.756 | 0.001 *** | 2.3907 | 0.010 ** |
|  | **Unpainted: Effects of 0.5X Grooming** (UP Controls and UP 0.5X; 16, 30d) | **Day** | 11.2216 | 0.001*** | 12.6313 | 0.001 *** | 10.3582 | 0.001*** |
|  |  | **Freq** | 3.1369 | 0.035* | 3.4022 | 0.035 * | 1.9044 | 0.078 |
|  |  | **Day*Freq** | 2.367 | 0.098 | 2.8002 | 0.068 | 1.6389 | 0.106 |
|  | **Unpainted: Effects of 3X Grooming** (UP Controls, UP 3X; 16, 30, 37d) | **Day** | 8.5804 | 0.001*** | 9.5868 | 0.001 *** | 8.3631 | 0.001*** |
|  |  | **Freq** | 8.8193 | 0.001*** | 7.8064 | 0.001 *** | 7.399 | 0.001*** |
|  |  | **Day*Freq** | 4.2046 | 0.001*** | 5.696 | 0.001 *** | 2.7949 | 0.008** |
|  | **Painted: Effects of all Grooming** (P Controls, P 0.5X and P 3X; 16, 30d, 37d) | **Day** | 7.864 | 0.001*** | 8.7629 | 0.001 *** | 9.9052 | 0.001 *** |
|  |  | **Freq** | 5.94 | 0.001*** | 6.4697 | 0.001 *** | 7.0816 | 0.001 *** |
|  |  | **Day*Freq** | 2.8844 | 0.002*** | 4.1142 | 0.001 *** | 2.7111 | 0.005 ** |
|  | **Painted: Effects of 0.5X Grooming** (P Controls and P 0.5X; 16, 30d) | **Day** | 7.2808 | 0.001*** | 7.1479 | 0.001 *** | 10.7636 | 0.001*** |
|  |  | **Freq** | 3.2536 | 0.014* | 4.1124 | 0.012 * | 3.9235 | 0.031 * |
|  |  | **Day*Freq** | 2.9037 | 0.027* | 4.2321 | 0.009 ** | 2.189 | 0.08 |
|  | **Painted: Effects of 3X Grooming** (P Controls and P 3X; 16, 30, 37d) | **Day** | 6.3154 | 0.001*** | 7.3267 | 0.001 *** | 7.5033 | 0.001*** |
|  |  | **Freq** | 9.0906 | 0.001*** | 7.0391 | 0.002 ** | 11.313 | 0.001*** |
|  |  | **Day*Freq** | 3.0416 | 0.004*** | 3.5256 | 0.004 ** | 3.1449 | 0.006** |
| **Press+ Pulse** | **Combined effect of Paint and all Grooming** (UP controls, P 0.5X and P 3X; 16, 30, 37d) | **Day** | 6.6619 | 0.001*** | 9.1129 | 0.001*** | 8.2931 | 0.001*** |
|  |  | **Freq** | 12.6237 | 0.001*** | 17.0524 | 0.001*** | 13.178 | 0.001*** |
|  |  | **Day*Freq** | 4.3959 | 0.001*** | 6.033 | 0.001*** | 3.898 | 0.001*** |
|  | **Combined effect of Paint and 0.5X Grooming** (UP Controls, P 0.5X; 16, 30d) | **Day** | 6.0702 | 0.002 ** | 8.3804 | 0.001*** | 8.3256 | 0.001*** |
|  |  | **Freq** | 11.7632 | 0.001 *** | 14.1673 | 0.001*** | 11.0413 | 0.001*** |
|  |  | **Day*Freq** | 5.1081 | 0.003 ** | 5.4595 | 0.002 ** | 3.9854 | 0.003 ** |
|  | **Combined effect of Paint and 0.5X Grooming** (UP Controls, P 3X; 16, 30, 37d) | **Day** | 5.2925 | 0.001*** | 6.4286 | 0.001*** | *6.0536* | *0.001**** |
|  |  | **Freq** | 21.1212 | 0.001*** | 26.8399 | 0.001*** | *22.4735* | *0.001**** |
|  |  | **Day*Freq** | 5.0044 | 0.001*** | 6.7368 | 0.001*** | 4.6086 | 0.001*** |

**Table S4**. PERMANOVA results for eukaryotic beta diversity. Bray-Curtis and Weighted Unifrac dissimilarities were calculated based on filtered proportion data. Unweighted Unifrac distances were calculated based on filtered rarefied (abundance) data. Results in italics are from data subsets with non-homogenous variances according to a betadisper test.

| **Dist- urbance** | **Effects tested** |  | **Bray-Curtis** | | **Weighted Unifrac** | | **Unweighted Unifrac** | |
| --- | --- | --- | --- | --- | --- | --- | --- | --- |
|  |  |  | **F** | **Pr(>F)** | **F** | **Pr(>F)** | **F** | **Pr(>F)** |
| **Global** | **All Samples: Effects of surface type and grooming** (UP controls, UP groomed, P controls and P Groomed, all freq: 16, 23, 30, 37d; permutations constrained by plate number) | **Day** | 6.1186 | 0.001 *** | 9.5282 | 0.001 *** | *10.8542* | *0.001 **** |
|  |  | **Type** | 13.3597 | 0.001 *** | *14.9911* | *0.001 **** | 10.8142 | 0.001 *** |
|  |  | **Freq** | 8.3843 | 0.001 *** | 14.7874 | 0.001 *** | 4.5939 | 0.002 *** |
|  |  | **Day*Type** | 4.2501 | 0.001 *** | 6.3033 | 0.001 *** | 2.7303 | 0.006 *** |
|  |  | **Day*Freq** | 3.3454 | 0.001 *** | 5.5148 | 0.001 *** | 2.1536 | 0.057 |
|  |  | **Type*Freq** | 4.7282 | 0.001 *** | 7.3759 | 0.001 *** | 4.9919 | 0.001 *** |
|  |  | **Day*Type***  **Freq** | 2.1437 | 0.07 | 2.8418 | 0.08 | 2.2128 | 0.056 |
| **Press** | **Controls: Effect of surface type** (UP controls and P control; 16, 23, 30, 37d) | **Day** | 2.286 | 0.001 *** | 3.2582 | 0.001 *** | 4.9771 | 0.001 *** |
|  |  | **Type** | 5.6049 | 0.001 *** | 6.3405 | 0.002 *** | 5.1459 | 0.001 *** |
|  |  | **Day*Type** | 1.9424 | 0.005 ** | 1.931 | 0.043 | 2.1681 | 0.009 ** |
| **Pulse** | **Unpainted: Effects of all Grooming** (UP Controls, UP 0.5X and UP 3X; 16, 30, 37d) | **Day** | 3.4912 | 0.001 *** | 4.3108 | 0.001 *** | 8.4617 | 0.001 *** |
|  |  | **Freq** | 6.7366 | 0.001 *** | 8.6103 | 0.001 *** | 3.8458 | 0.002 ** |
|  |  | **Day*Freq** | 2.749 | 0.002 *** | 4.7714 | 0.001 *** | 2.6767 | 0.002 ** |
|  | **Unpainted: Effects of 0.5X Grooming** (UP Controls and UP 0.5X; 16, 30d) | **Day** | 2.6987 | 0.001 *** | 3.8152 | 0.025 * | 10.22 | 0.001 *** |
|  |  | **Freq** | 1.91 | 0.023 * | 4.5903 | 0.013 * | 2.2708 | 0.073 |
|  |  | **Day*Freq** | 2.0103 | 0.022 * | 6.9656 | 0.002 ** | 2.1596 | 0.075 |
|  | **Unpainted:** **Effects of 3X Grooming** (UP Controls, UP 3X; 16, 30, 37d) | **Day** | 3.4106 | 0.001 *** | 4.5216 | 0.001 *** | 7.1348 | 0.001 *** |
|  |  | **Freq** | 10.2884 | 0.001 *** | 14.0529 | 0.001 *** | 5.0433 | 0.001 *** |
|  |  | **Day*Freq** | 3.0687 | 0.001 *** | 5.6839 | 0.001 *** | 2.8394 | 0.002 ** |
|  | **Painted: Effects of all Grooming** (P Controls, P 0.5X and P 3X; 16, 30d, 37d) | **Day** | 6.3535 | 0.001 *** | 10.117 | 0.001 *** | 5.5266 | 0.001 *** |
|  |  | **Freq** | 7.2749 | 0.001 *** | 14.1499 | 0.001 *** | 5.5009 | 0.001 *** |
|  |  | **Day*Freq** | 2.7649 | 0.005 ** | 4.0949 | 0.003 ** | 1.6786 | 0.043 * |
|  | **Painted: Effects of 0.5X Grooming (**P Controls and P 0.5X; 30d) | **Freq** | *4.1505* | *0.1* | *9.7511* | *0.1* | 2.1231 | 0.1 |
|  | **Painted**: **Effects of 3X Grooming** (P Controls and P 3X; 30, 37d) | **Day** | *2.1586* | *0.077* | *2.376* | *0.092* | 3.4608 | 0.010 ** |
|  |  | **Freq** | *8.7336* | *0.002 *** | *18.962* | *0.002 **** | 9.3448 | 0.001 *** |
|  |  | **Day*Freq** | *2.0291* | *0.099* | *2.504* | *0.109* | 1.6442 | 0.107 |
| **Press+ Pulse** | **Combined effect of Paint and all Grooming** (UP controls, P 0.5X and P 3X; 16, 30, 37d) | **Day** | 5.5157 | 0.001 *** | 13.5291 | 0.001 *** | 6.6151 | 0.001 *** |
|  |  | **Freq** | 8.9814 | 0.001 *** | 15.3355 | 0.001 *** | 6.6168 | 0.001 *** |
|  |  | **Day*Freq** | 4.2032 | 0.001 *** | 7.4464 | 0.001 *** | 2.8469 | 0.001 *** |
|  | **Combined effect of Paint and 0.5X Grooming (**UP Controls, P 0.5X; 16, 30d) | **Day** | 5.2551 | 0.002 ** | 20.752 | 0.001 *** | 6.8204 | 0.001 *** |
|  |  | **Freq** | 6.7377 | 0.001 *** | 20.073 | 0.001 *** | 4.3781 | 0.001 *** |
|  |  | **Day*Freq** | 5.215 | 0.002 ** | 16.461 | 0.001 *** | 3.1896 | 0.009 ** |
|  | **Combined effect of Paint and 0.5X Grooming** (UP Controls, P 3X; 16, 30, 37d) | **Day** | 3.7699 | 0.001 *** | 9.7856 | 0.001 *** | 5.6027 | 0.001 *** |
|  |  | **Freq** | 12.476 | 0.001 *** | 20.4171 | 0.001 *** | 11.0596 | 0.001 *** |
|  |  | **Day*Freq** | 3.8733 | 0.001 *** | 6.0796 | 0.002 ** | 3.3067 | 0.001 *** |

**
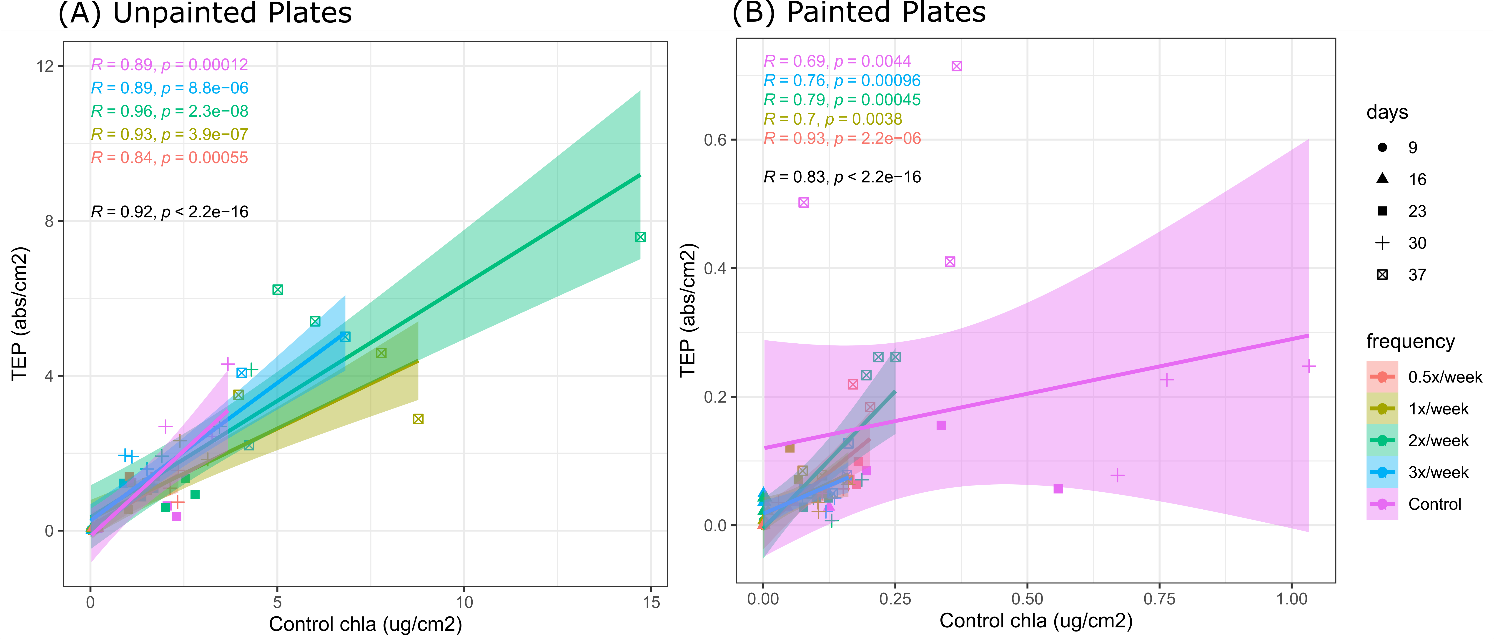
**

**Figure S1.** Spearman Correlation between Chl *a* (Kamensky et al., 2020) and TEP concentrations in the biofilms on (A) Unpainted and (B) Painted plates


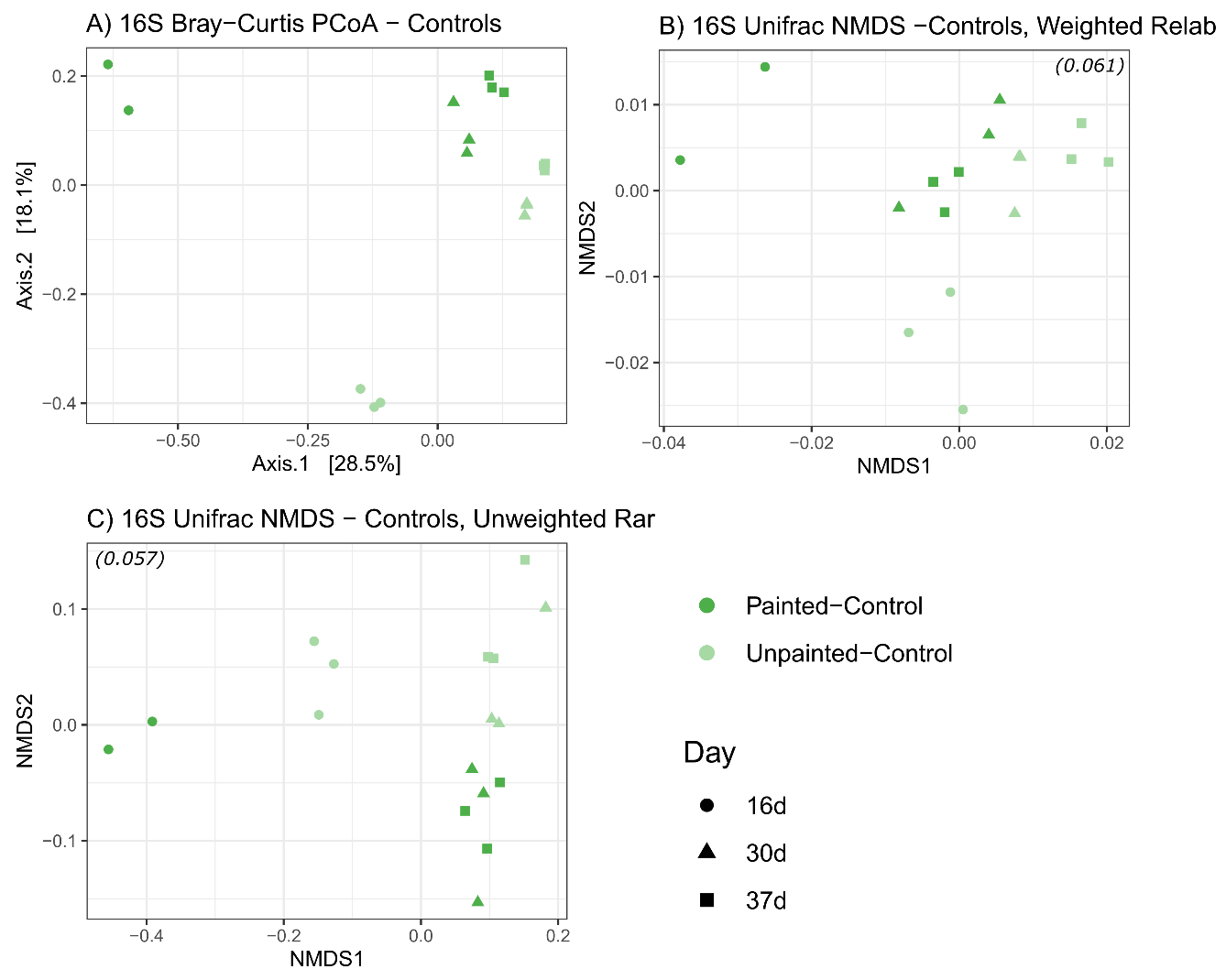


**Figure S2.** ASV-level beta-diversity of control (ungroomed) bacterial communities growing on Unpainted and Painted plates. (A) PCoA of Bray-Curtis dissimilarities calculated using relative abundances. (B) NMDS of Weighted Unifrac dissimilarities calculated using relative abundances. (C) NMDS of Unweighted Unifrac dissimilarities calculated using rarefied counts (“Rar”). On the NMDS plots, italicized values in parentheses represent stress.


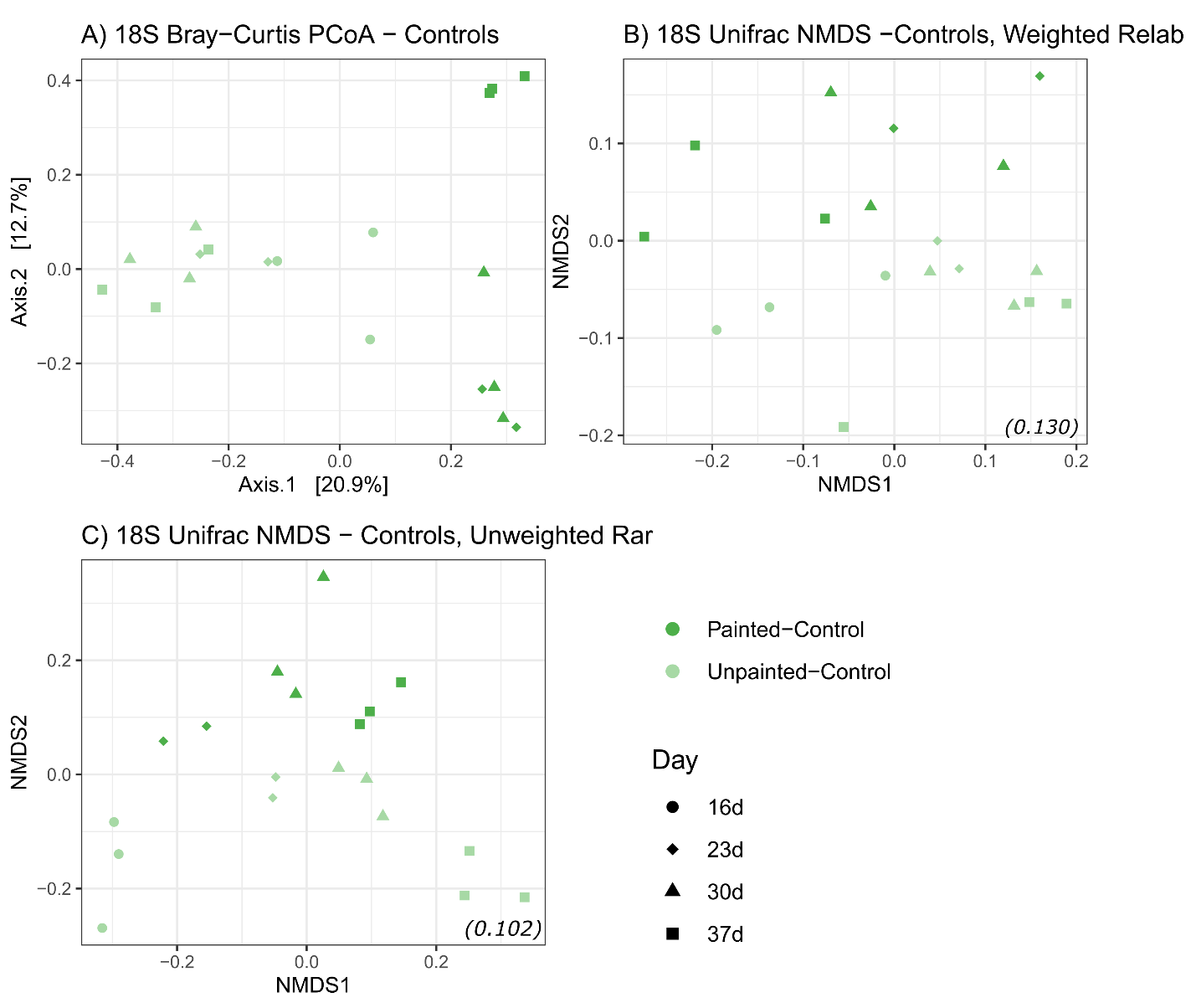


**Figure S3.** ASV-level beta-diversity of control (ungroomed) eukaryotic communities growing on Unpainted and Painted plates. (A) PCoA of Bray-Curtis dissimilarities calculated using relative abundances. (B) NMDS of Weighted Unifrac dissimilarities calculated using relative abundances. (C) NMDS of Unweighted Unifrac dissimilarities calculated using rarefied counts (“Rar”). On the NMDS plots, italicized values in parentheses represent stress.


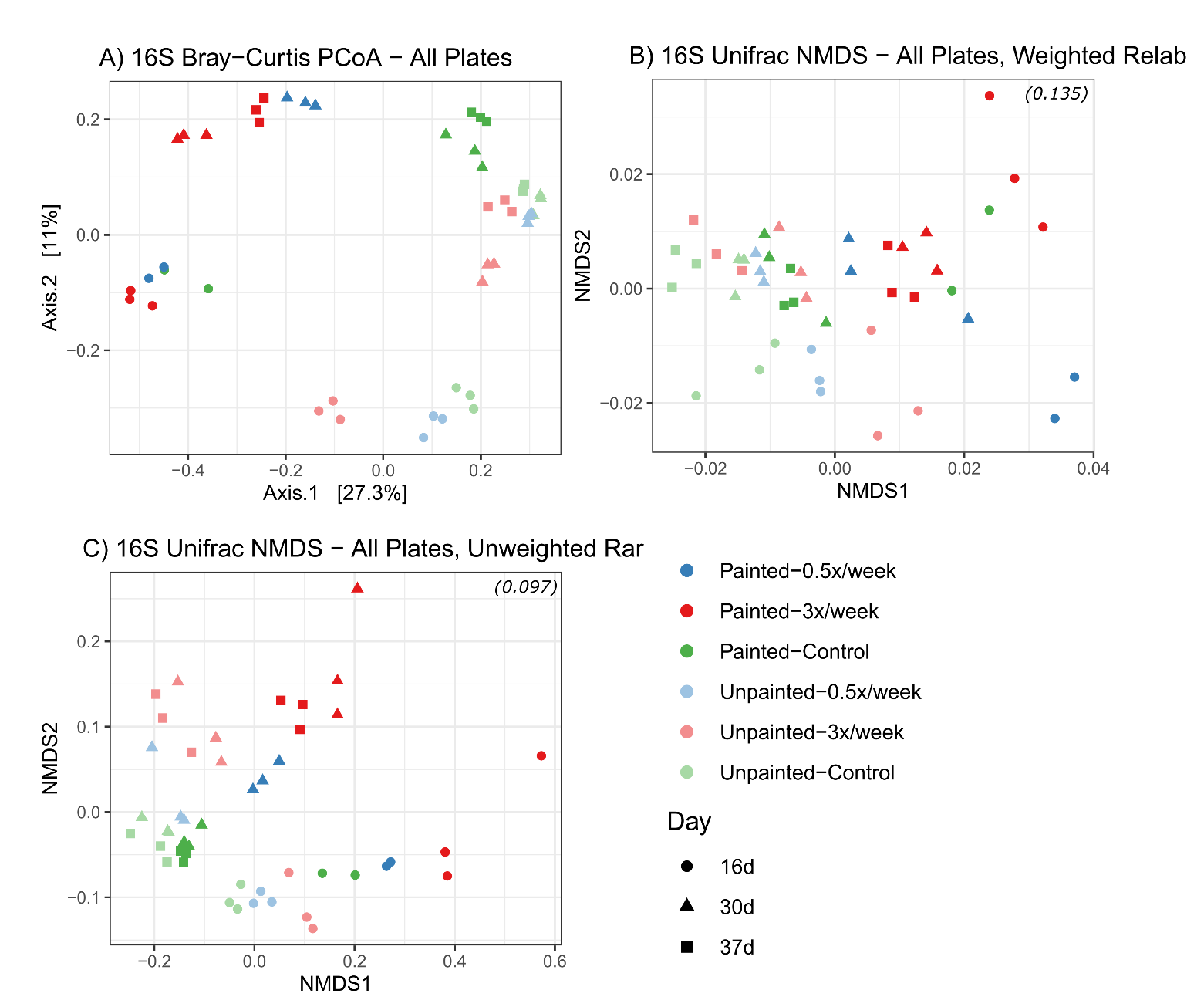


**Figure S4.** ASV-level beta-diversity of all bacterial communities growing on Unpainted and Painted plates. (A) PCoA of Bray-Curtis dissimilarities calculated using relative abundances. (B) NMDS of Weighted Unifrac dissimilarities calculated using relative abundances. (C) NMDS of Unweighted Unifrac dissimilarities calculated using rarefied counts (“Rar”). On the NMDS plots, italicized values in parentheses represent stress.


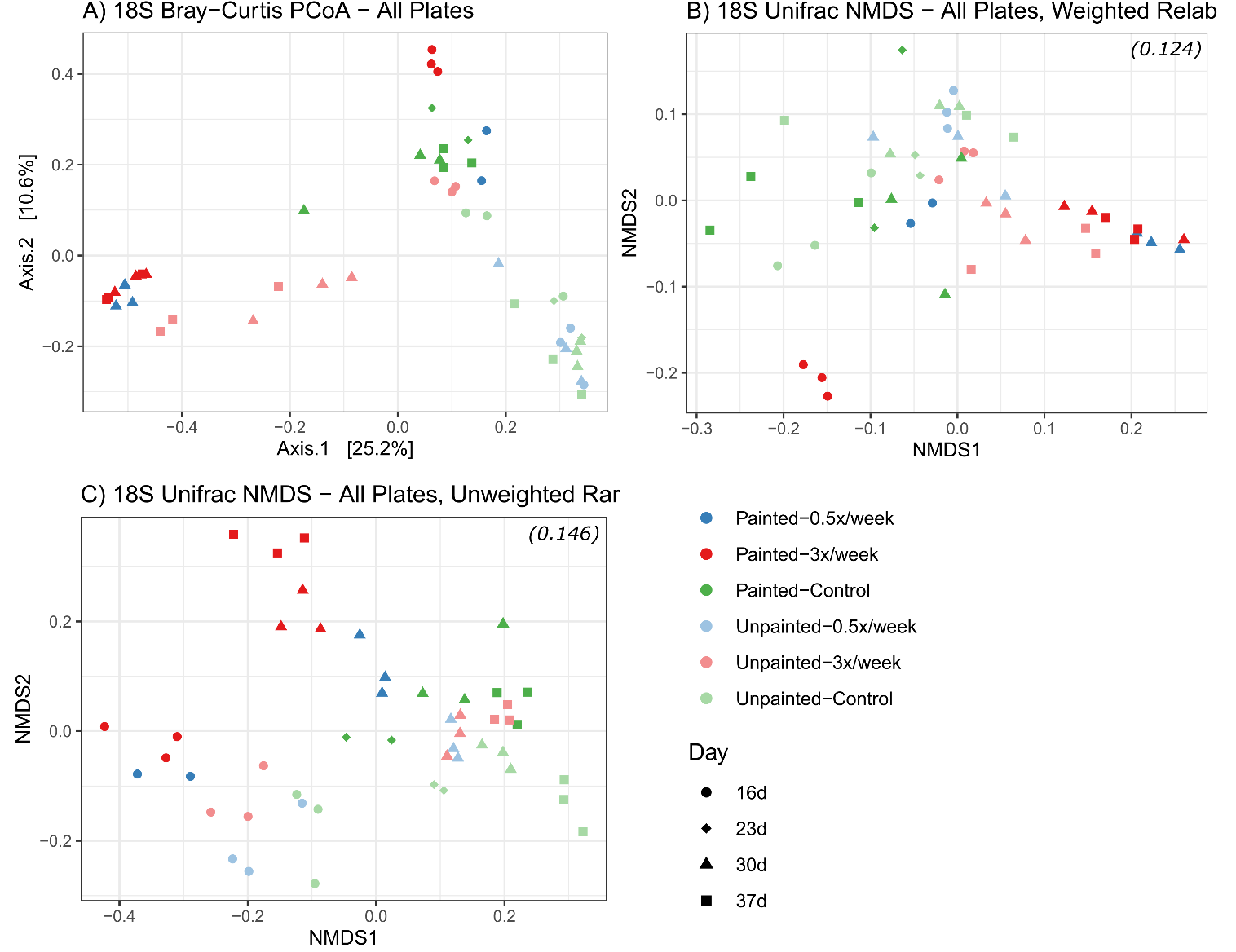


**Figure S5.** ASV-level beta-diversity of all eukaryotic communities on Unpainted and Painted plates. (A) PCoA of Bray-Curtis dissimilarities calculated using relative abundances. (B) NMDS of Weighted Unifrac dissimilarities calculated using relative abundances. (C) NMDS of Unweighted Unifrac dissimilarities calculated using rarefied counts (“Rar”). On the NMDS plots, italicized values in parentheses represent stress.
